# Supplementary material for: Ultrastable and efficient slight-interlayer-displacement 2D Dion-Jacobson perovskite solar cells
Source: Nat Commun. 2024 Jul 8;15:5709. doi: 10.1038/s41467-024-50018-4 (PMC11231157; doi:10.1038/s41467-024-50018-4)
Supplement: Supplementary file 1 — Supplementary Information [file 41467_2024_50018_MOESM1_ESM.pdf]

Supplementary information for

Ultrastable slight-interlayer-displacement Dion−Jacobson perovskite solar cells

Weichuan Zhang[1,2,3], Ziyuan Liu[4], Lizhi Zhang[4], Hui Wang [4], Chuanxiu Jiang[3,5], Xianxin Wu[3,5], Chuanyun Li[1,6], Shengli Yue[7], Rongsheng Yang[1], Hong Zhang[1], Jianqi Zhang[1], Xinfeng Liu[3,5], Yuan Zhang[7] & Huiqiong Zhou [1,3]*

[1] CAS Key Laboratory of Nano system and Hierarchical Fabrication, National Center for Nanoscience and Technology, Beijing 100190, People's Republic of China

[2] School of Electrical Engineering, University of South China, Hengyang, Hunan 421001, PR China

[3] University of Chinese Academy of Sciences, Beijing 100049, People's Republic of China

[4] Laboratory of Theoretical and Computational Nanoscience, National Center for Nanoscience and Technology, Chinese Academy of Sciences, Beijing 100190, China

[5] CAS Key Laboratory of Standardization and Measurement for Nanotechnology, National Center for Nanoscience and Technology, Beijing 100190, P. R. China

[6] College of Chemistry and Materials Engineering, Beijing Technology And Business University, Beijing, 100048, P. R. China

[7] Beijing Advanced Innovation Center for Biomedical Engineering, Beihang University, Beijing 100191, People's Republic of China

* Email: zhouhq@nanoctr.cn

Table of Contents

Supplementary Figures 3
Supplementary Tables 43
Supplementary References 62

Supplementary Figures

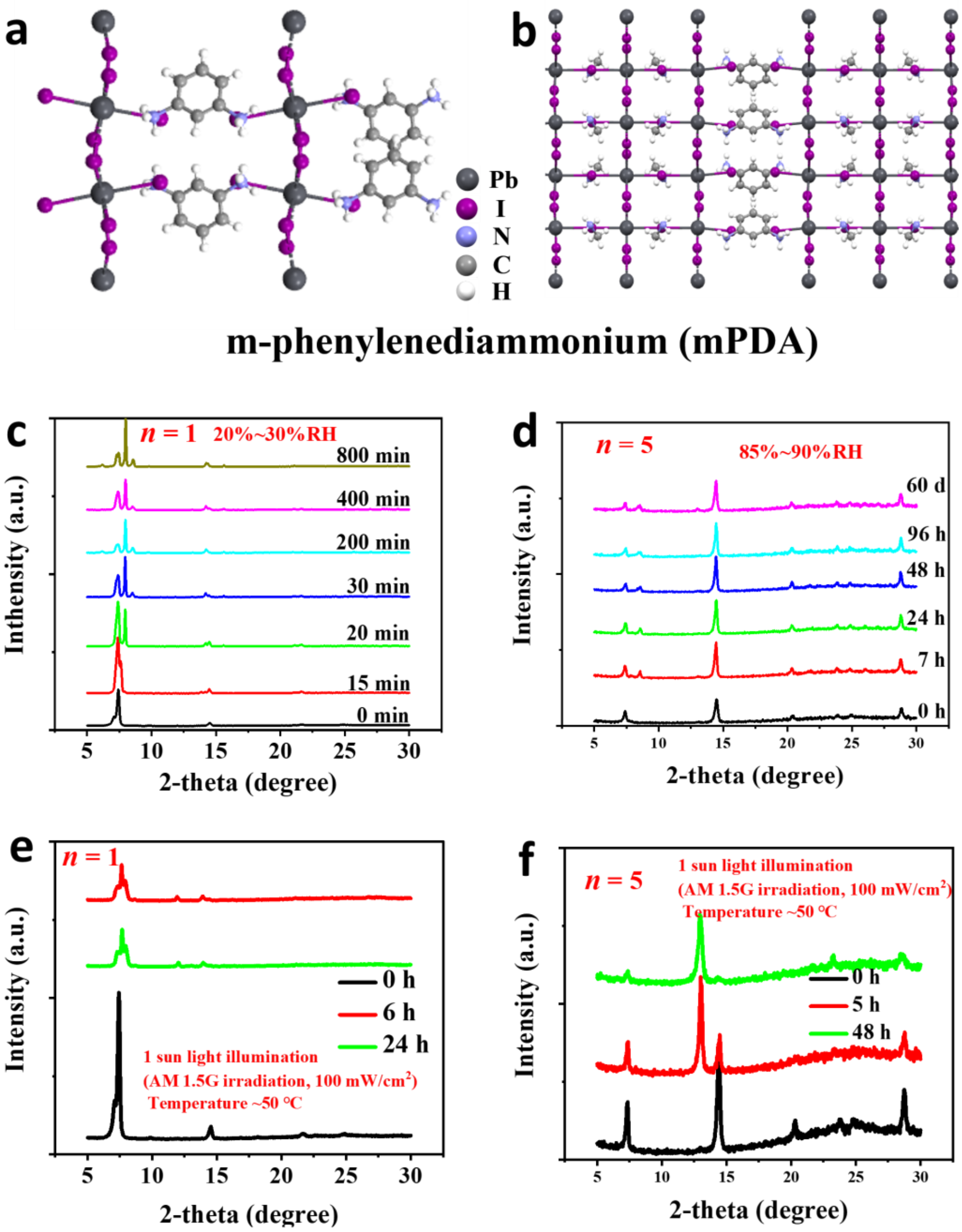

Supplementary Fig. 1 | Schematic diagrams of structure and XRD characterization. Schematic structural diagrams of the *m*-phenylenediammonium-based (mPDA) perovskites. **a** $n = 1$. **b** $n = 5$. **c**, **d** XRD patterns of $n = 1$ and $n = 5$ perovskite films for the humidity stability test in a constant temperature and humidity chamber, respectively. **e**, **f** $n = 1$ and $n = 5$ perovskite films for the light and thermal stability test in a glovebox with a 1-sun light and heating atmosphere, respectively.

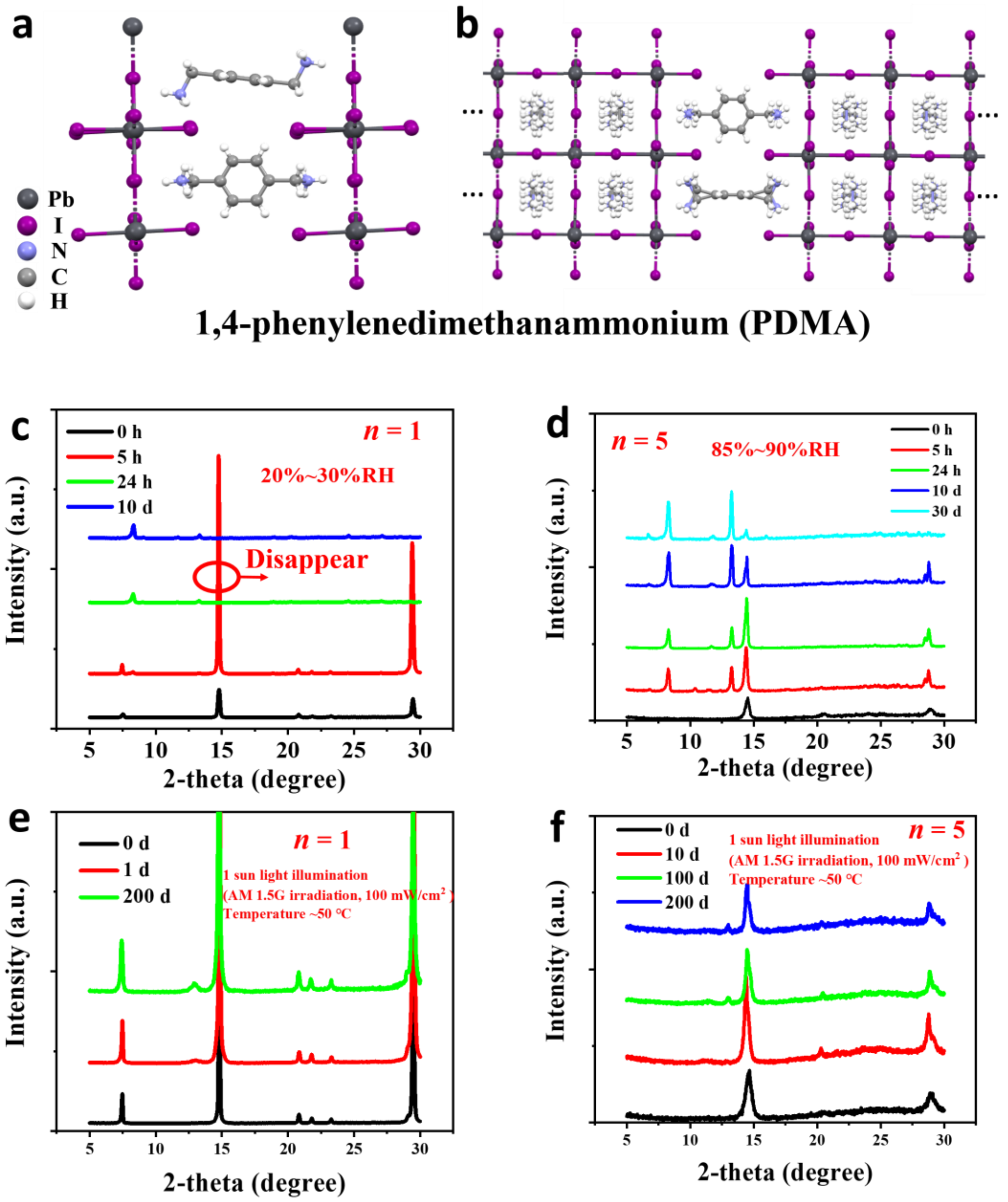

Supplementary Fig. 2 | Schematic diagrams of structure and XRD characterization. Schematic structural diagrams of the 1,4-phenylenedimethanammonium-based (PDMA) perovskites. **a** $n = 1$. **b** $n = 2$. **c**, **d** XRD patterns of $n = 1$ and $n = 5$ perovskite films for the humidity stability test in a constant temperature and humidity chamber, respectively. **e**, **f** $n = 1$ and $n = 5$ perovskite films for the light and thermal stability test in a glovebox with a 1-sun light and heating atmosphere, respectively.

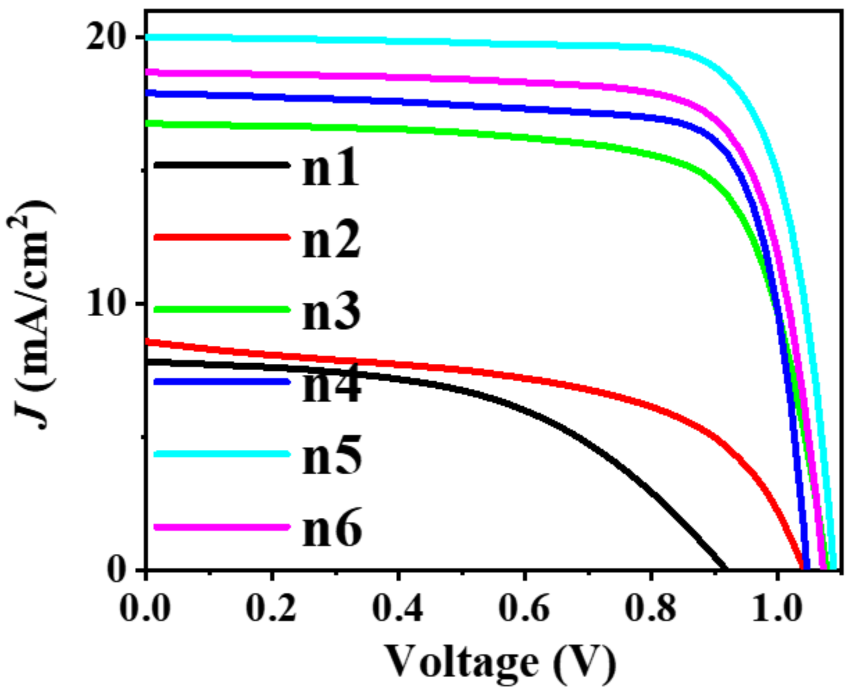

Supplementary Fig. 3 | Device performance of perovskite solar cells. *J*-*V* curves of the CDMA-based perovskite solar cells with different *n*-value active layers.

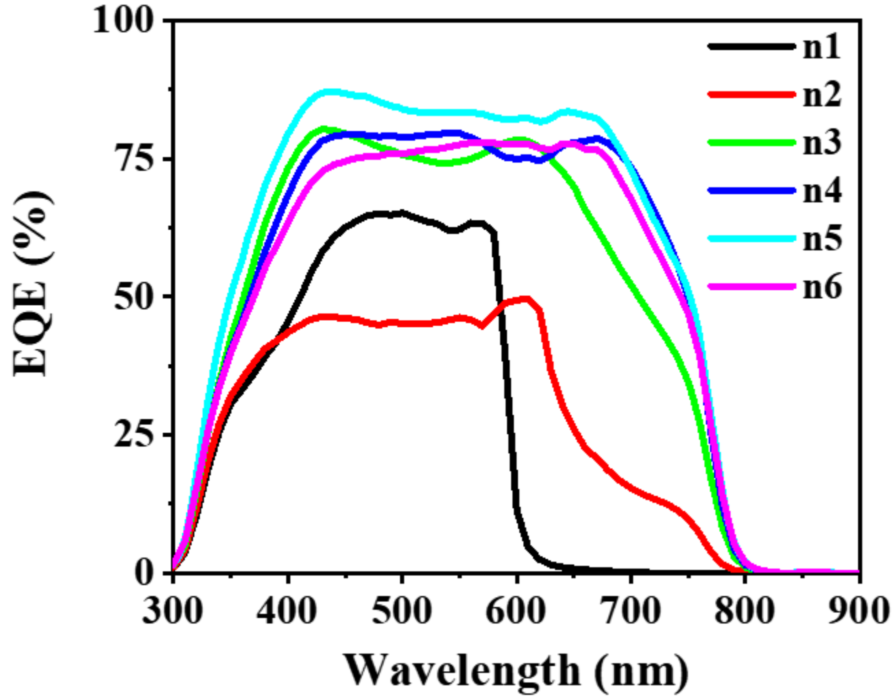

Supplementary Fig. 4 | Device performance of perovskite solar cells. EQE spectra of the CDMA-based perovskite solar cells with different *n*-value active layers.

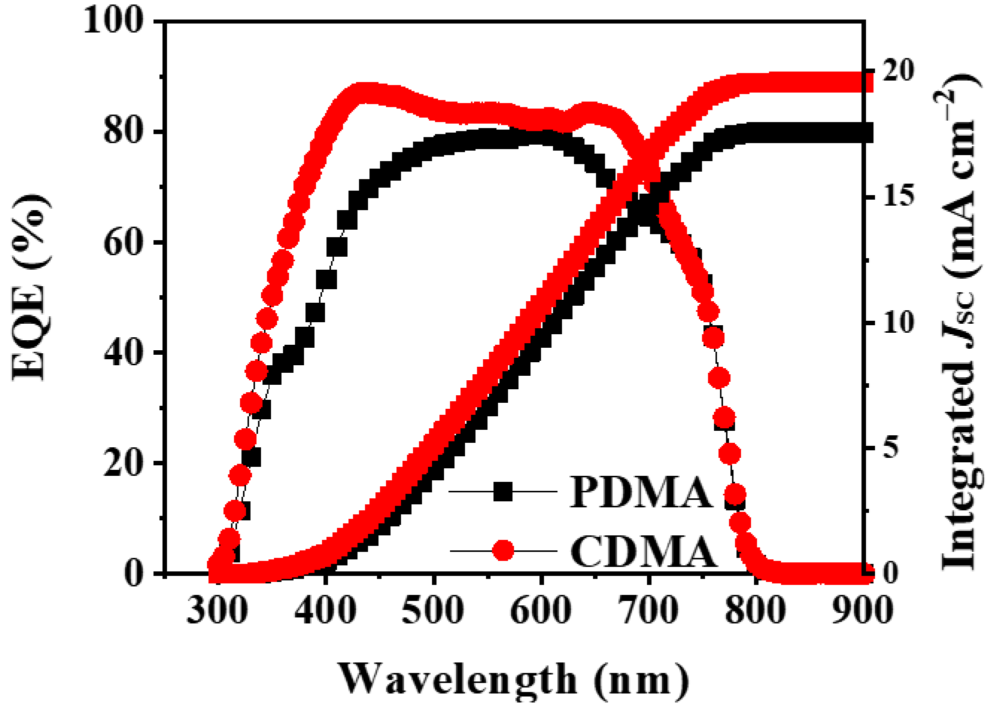

Supplementary Fig. 5 | Device performance of perovskite solar cells. EQE spectra of the PDMA- and CDMA-based perovskite solar cells.

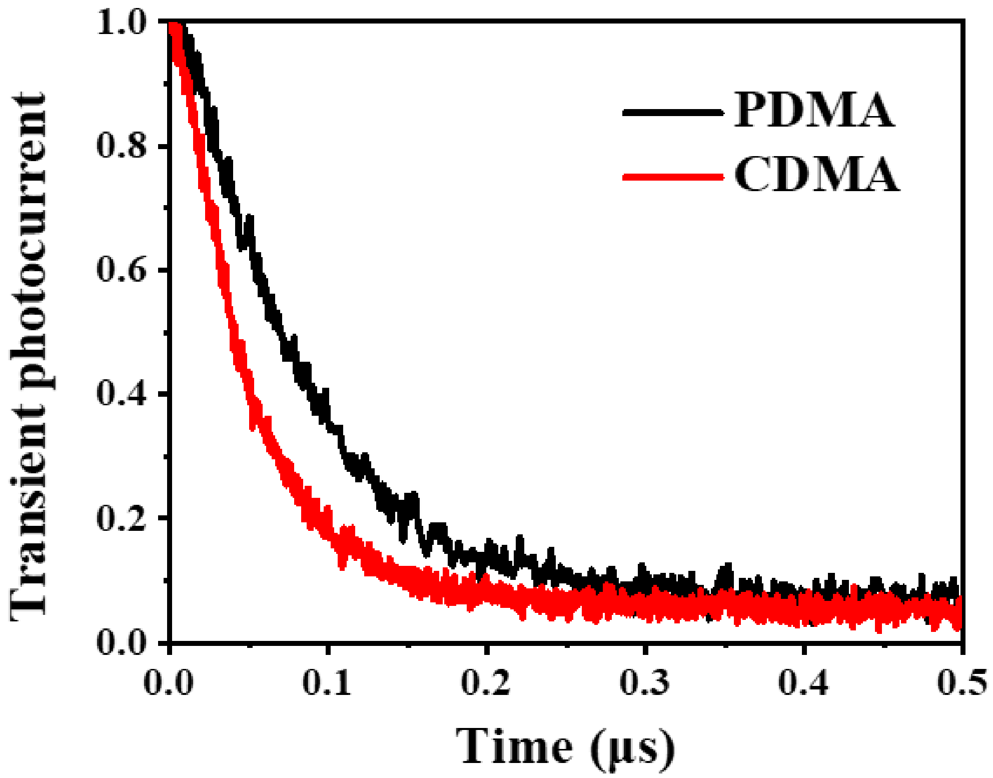

Supplementary Fig. 6 | Device performance of perovskite solar cells. Photocurrent decay curves of the corresponding devices.

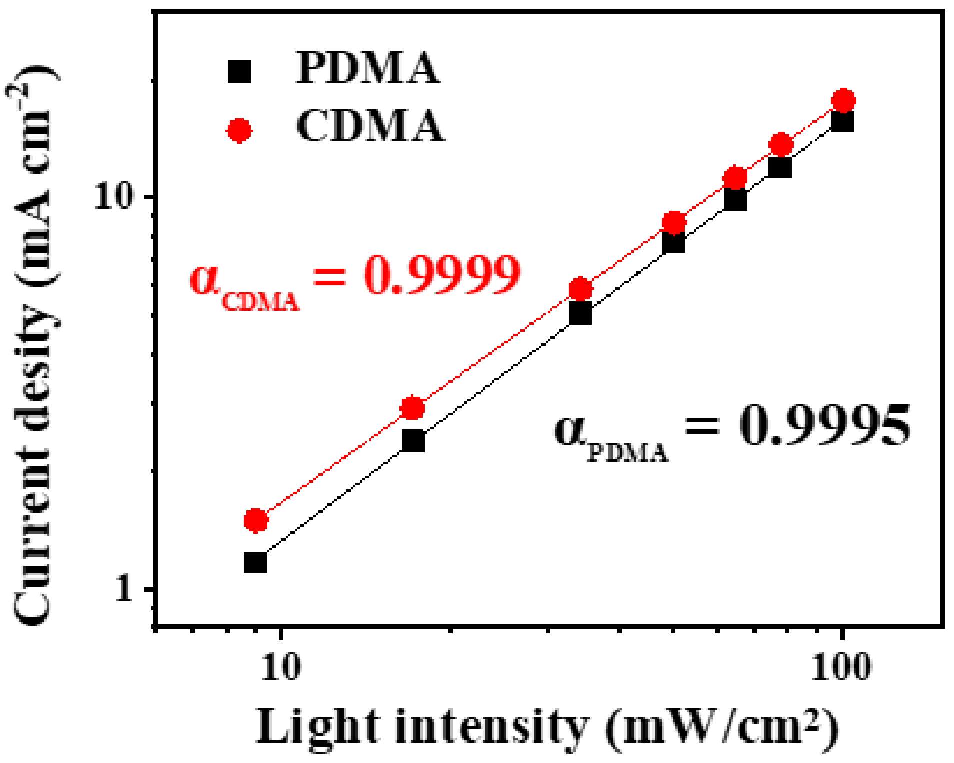

Supplementary Fig. 7 | Device performance of perovskite solar cells. Light-dependent J_{SC} of the 2D perovskite solar cells.

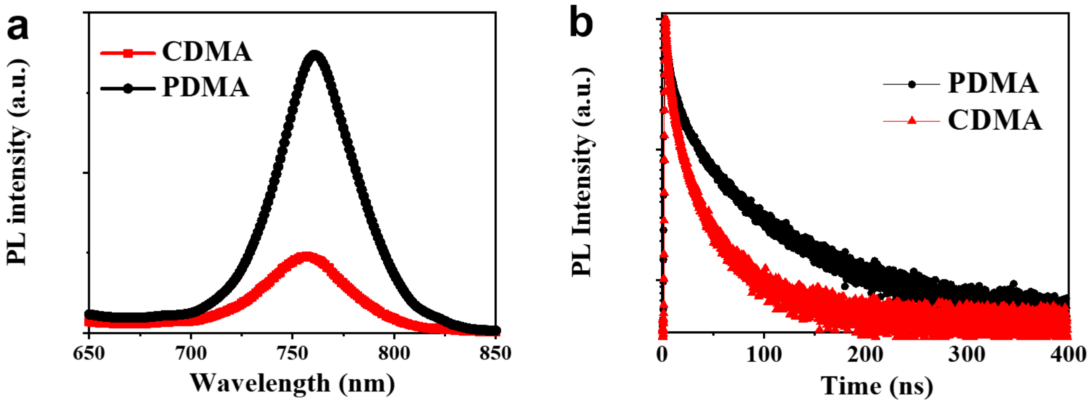

Supplementary Fig. 8 | Photoluminescence properties. a PL spectra of the DJ perovskite films deposited on PTAA transport layer substrates. **b** Time-resolved PL of the DJ perovskite films deposited on PTAA transport layer substrates.

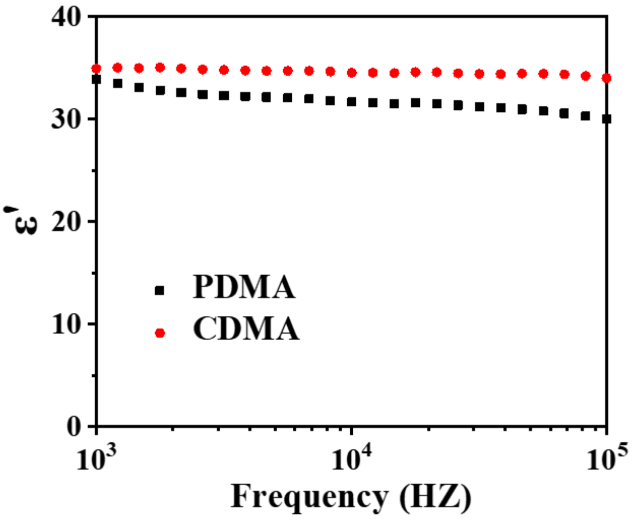

Supplementary Fig. 9 | Impedance measurements of perovskite devices. Dielectric constant versus frequency characteristics of PDMA- and CDMA-based perovskite devices determined by impedance measurements.

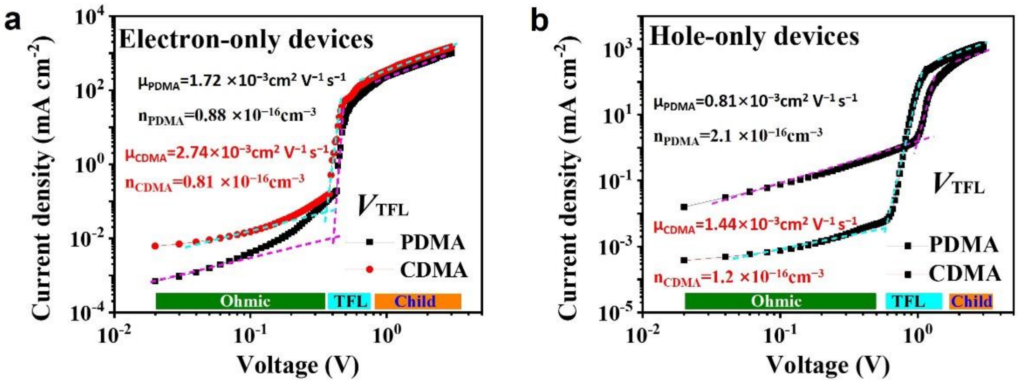

Supplementary Fig. 10 | Dark *J–V* curves of the PDMA- and CDMA-based perovskite devices. a Electron-only devices. **b** Hole-only devices.

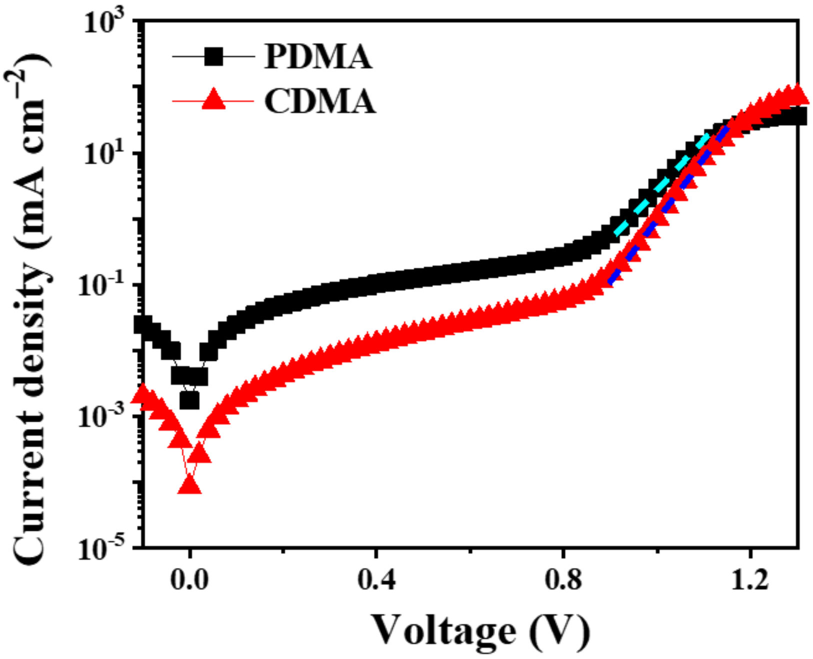

Supplementary Fig. 11 | Device performance of perovskite solar cells. Dark *J–V* curves for different perovskite devices.

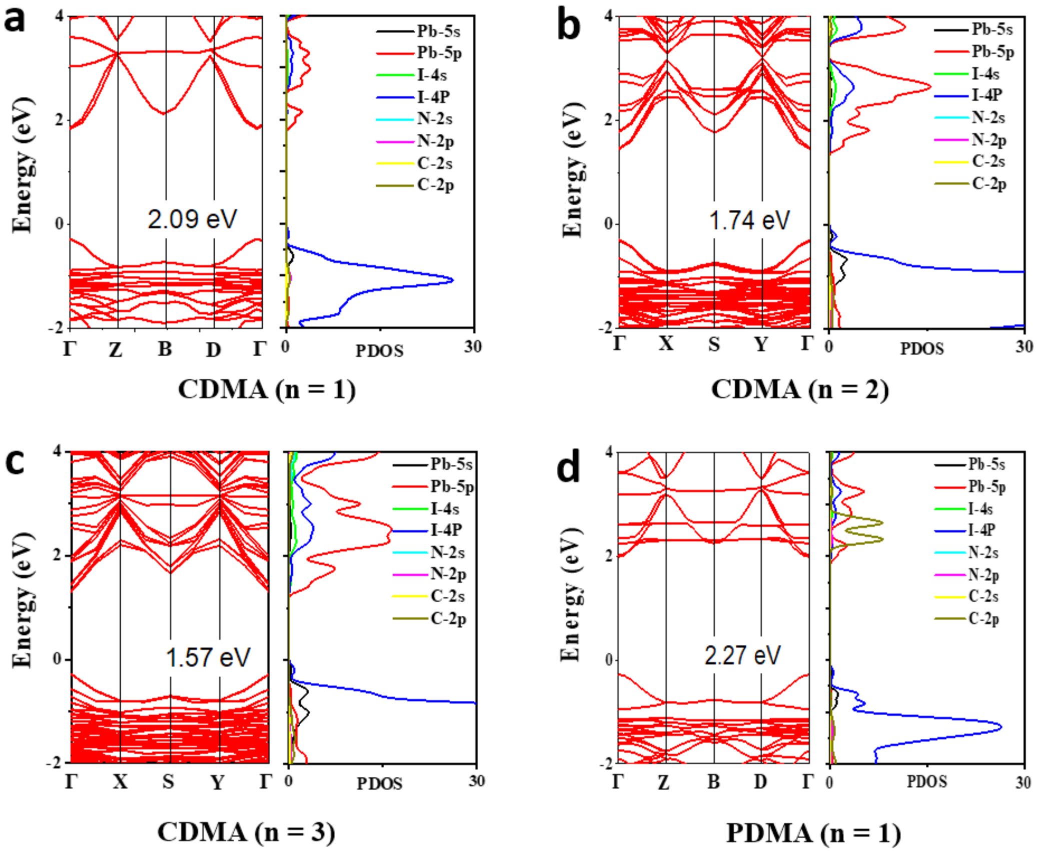

Supplementary Fig. 12 | Calculated band structure and projected density of states (PDOS, right). a Calculated result of the *n* =1 CDMA-based DJ perovskite. **b** Calculated result of the *n* =2 CDMA-based DJ perovskite. **c** Calculated result of the *n* =3 CDMA-based DJ perovskite. **d** Calculated result of the *n* =1 PDMA-based DJ perovskite. The electronic structure of all models was investigated using density functional theory. The calculations were performed with the Vienna ab initio Simulation Package (VASP)[1] using projector augmented-wave (PAW) potentials within the PBE exchange-correlation functional.[2] The supercell model of crystals was based on the experimental results. The lattice parameters and the internal atomic positions were fixed. The energy plane-wave cutoff was 500 eV. Monkhorst–Pack mesh[3] was used to sample the Brillouin zone with the KP-resolved value of 0.040. The density of states and band structures of all crystal structures were calculated.

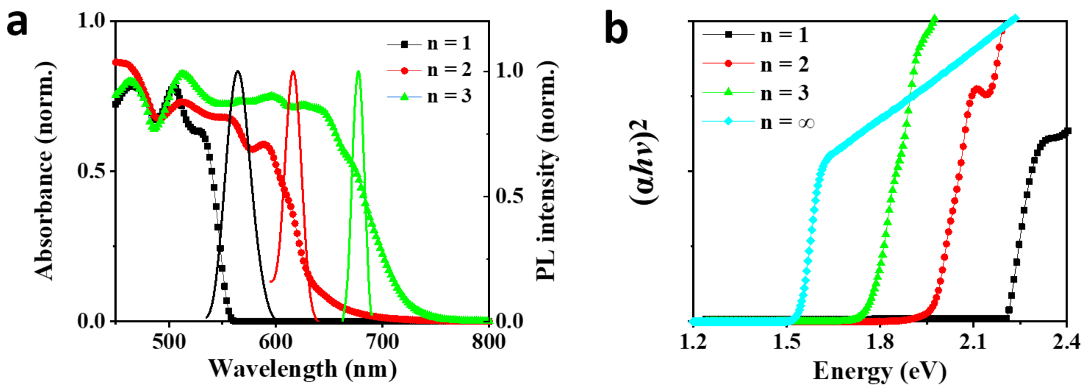

Supplementary Fig. 13 | Optical properties of perovskite crystals. a Optical absorption spectra and steady-state PL spectra. **b** Comparison of the bandgaps for the DJ series and 3D $MAPbI_3$ perovskite, showing great potential as the light-absorbing materials.

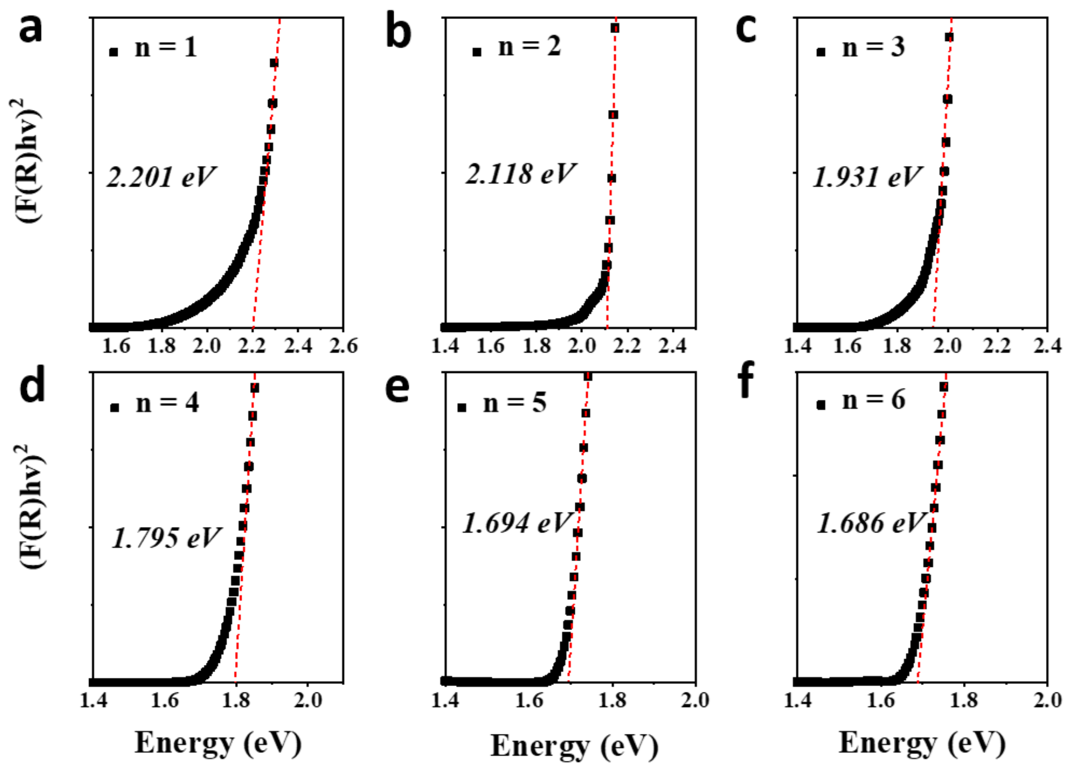

Supplementary Fig. 14 | Tauc plots of perovskite films. a *n* =1. **b** *n* = 2. **c** *n* = 3. **d** *n* = 4. **e** *n* = 5. **f** *n* = 6.

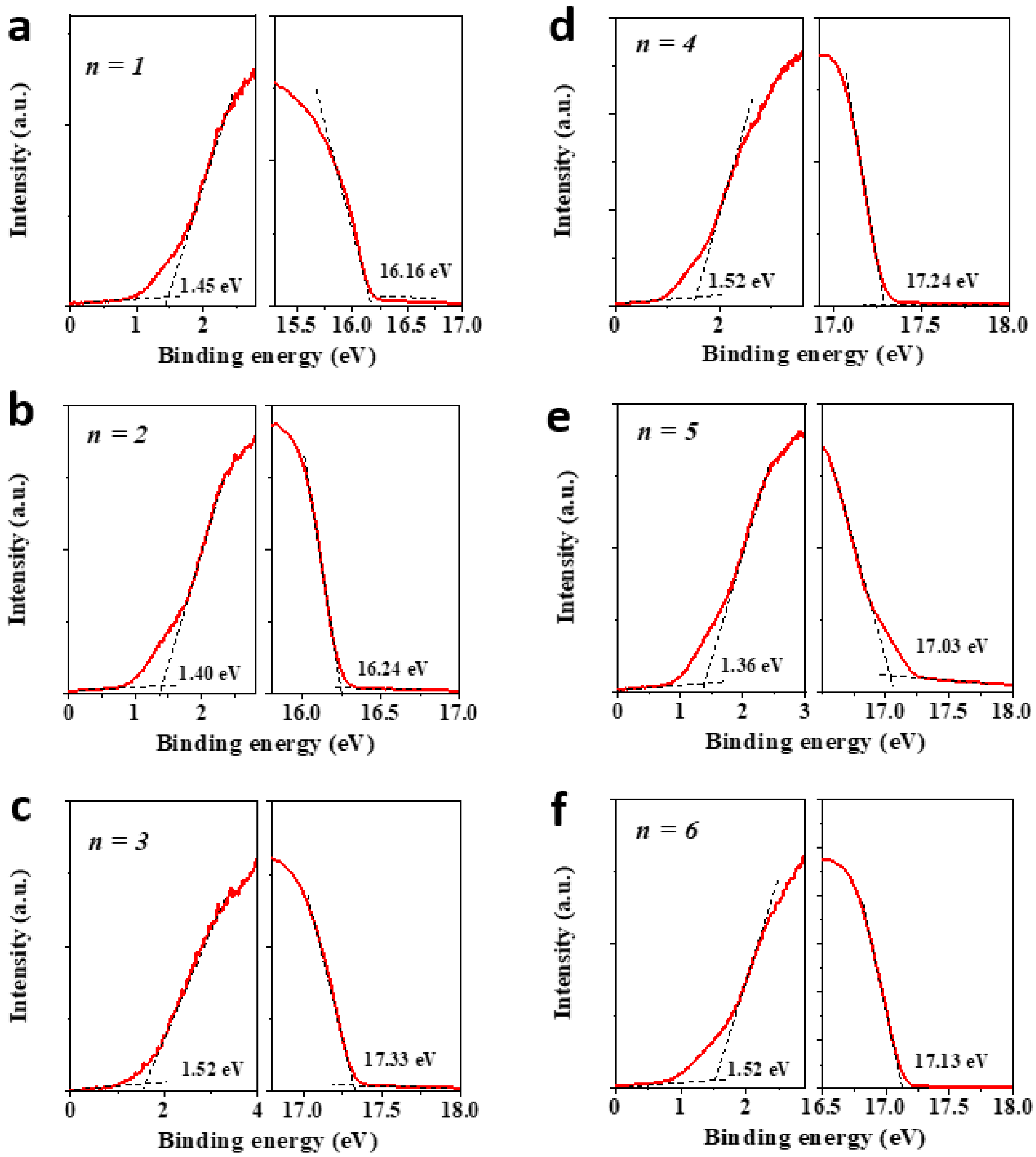

Supplementary Fig. 15 | The cutoff and onset energy region of UPS spectra for the DJ perovskite films. a *n* = 1. **b** *n* = 2. **c** *n* = 3. **d** *n* = 4. **e** *n* = 5. **f** *n* = 6.

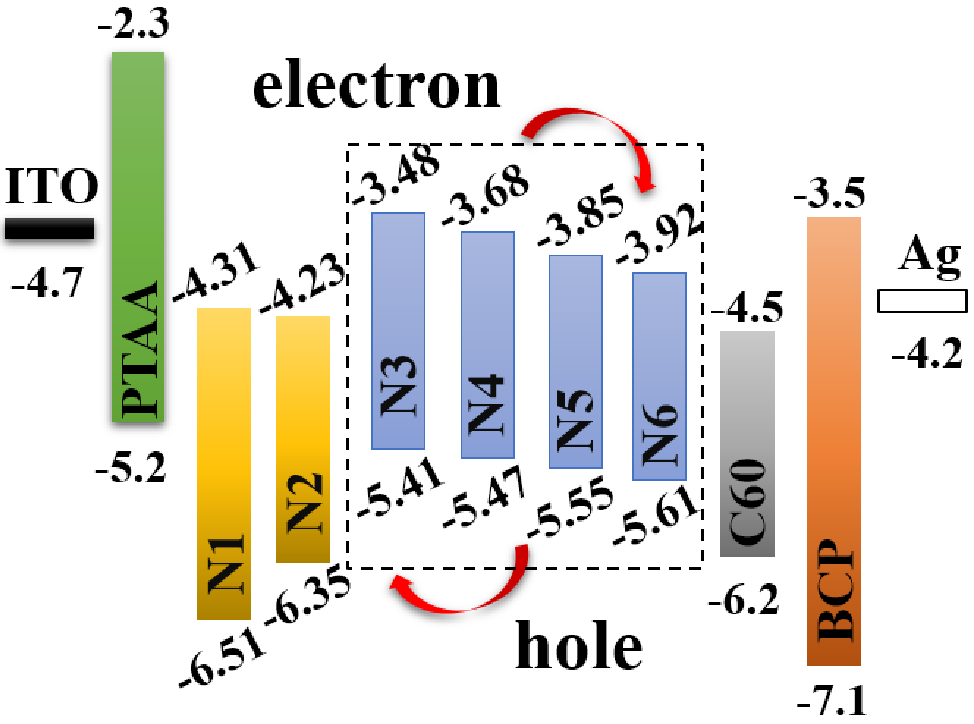

Supplementary Fig. 16 | Energy-level alignment diagram. Energy diagram of CDMA-based DJ series with different *n* values, deduced from Tauc plots and UPS measurements. Note: The graph was obtained without considering the exciton binding energy since the resulted perovskites (especially with large *n* values) are composited by mixed phases with different *n* values.

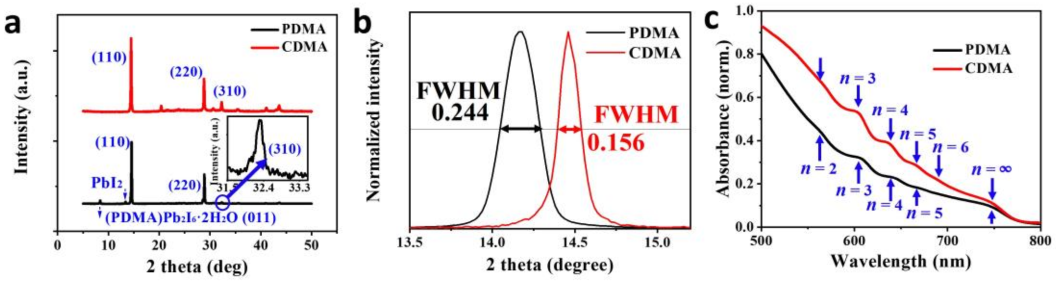

Supplementary Fig. 17 | Characterization of the perovskite films. a XRD patterns of the DJ perovskite films. **b** Comparison of FWHM (full width half maximum) of the (110)-oriented XRD peaks. **c** UV–vis absorption spectra of the perovskite films.

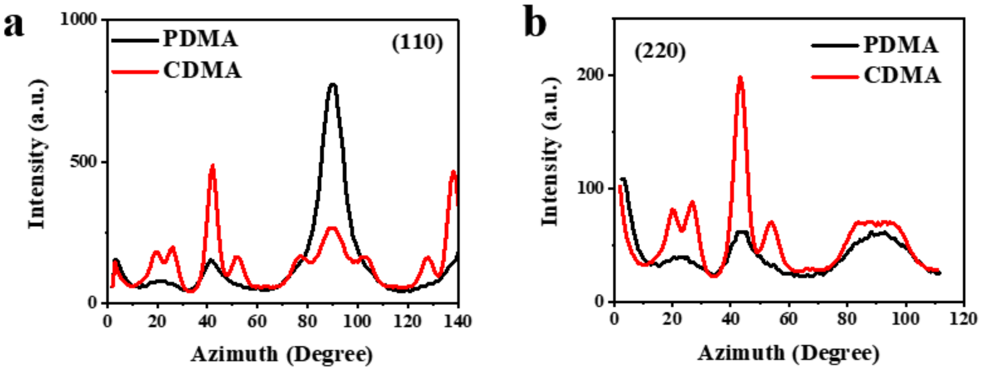

Supplementary Fig. 18 | Azimuthal scans from GIWAXS measurements. **a** (110) orientation. **b** (220) orientation.

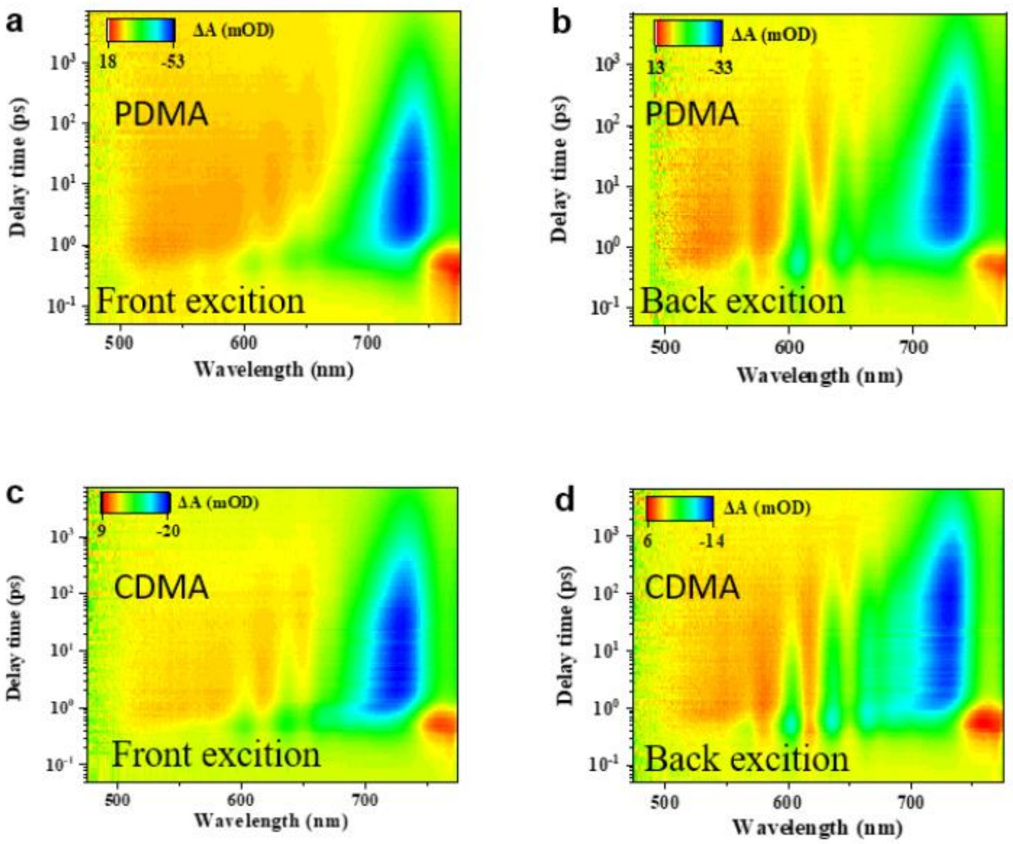

Supplementary Fig. 19 | TA color map of the reference 2D perovskite film. a PDMA films excited at front side. **b** PDMA films excited at back side. **c** CDMA films excited at front side. **d** CDMA films excited at back side.

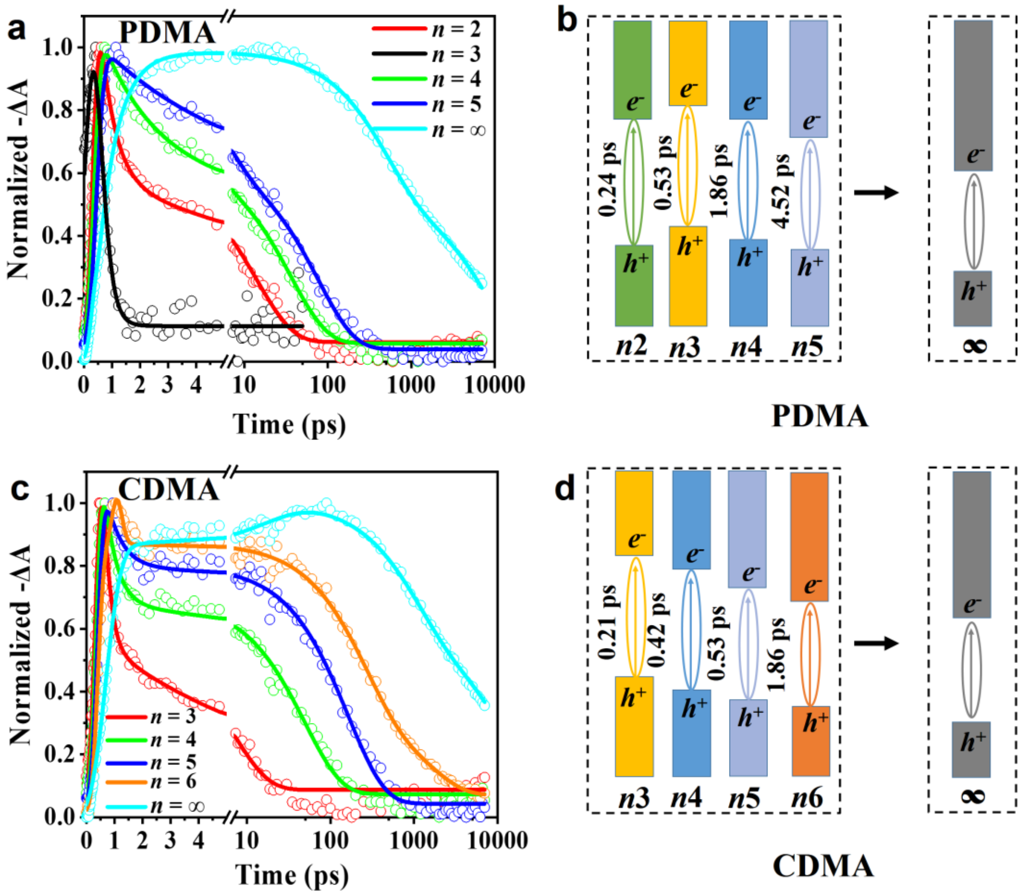

Supplementary Fig. 20 | Carrier transport pathway and schematic of the band structure for DJ perovskite QWs. a TA kinetics of PDMA-based perovskite film probed at n = 2, 3, 4, 5 and n ≈ ∞ bands. Solid lines are the fits of the kinetics by exponential function. **b** Schematic of the band structure for PDMA-based DJ perovskites and the corresponding decay times. **c** TA kinetics of CDMA-based perovskite film probed at n = 3, 4, 5, 6 and n ≈ ∞ bands. Solid lines are the fits of the kinetics by exponential function. **d** Schematic of the band structure for PDMA-based DJ perovskites and the corresponding decay times.

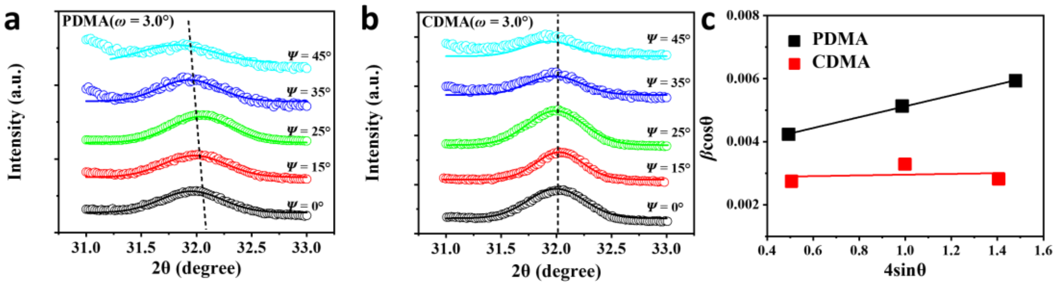

Supplementary Fig. 21 | Residual strain distribution measurement with the GIXRD method. a GIXRD spectrum at different tilt angles for the strain-free film of the PDMA DJ perovskite film (n = 5). **b** GIXRD spectrum at different tilt angles for the mixed-compressive-tensile-strained film of the CDMA perovskite film (n = 5). It reveals that the CDMA DJ series has a notable strain-free strain feature, which can contribute to the commercial scale. **c** Williamson−Hall plots fitting of DJ perovskite films. Based on the grain size and crystallinity analysis, Williamson–Hall plots were further applied to calculate the residual strain in perovskite films using Equation: $\beta cos\theta = \varepsilon(4sin\theta) + k\lambda/D$, where β is total broadening of XRD peaks, defined as FWHM, and θ is diffraction angle, ε is residual strain, K is Scherer constant (about 0.9 for perovskite), λ is wavelength of X-ray (1.5406 Å), and D is crystal size of perovskite film. The detailed linear fitting and data are shown in Supplementary Fig. 21c. The fit value of the calculated residual strain further shows an almost free residual strain of the CDMA films, consistent with the depth-dependent GIXRD measurements.

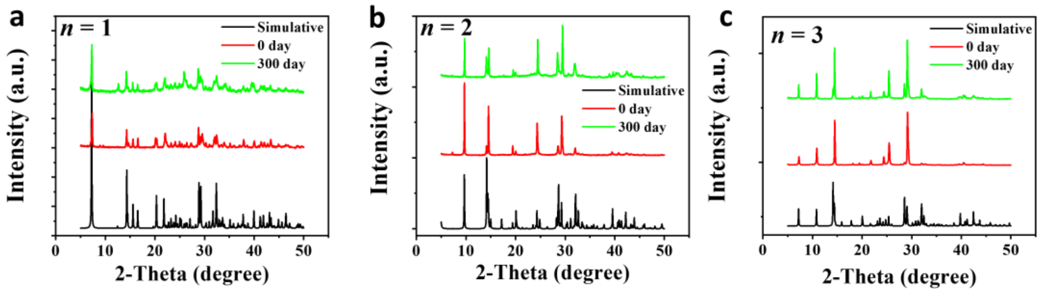

Supplementary Fig. 22 | XRD patterns. XRD patterns of *n* =1-3 CDMA-based DJ perovskite power sample measured by the pure powder samples, showing an excellent material stability over 300 days under the atmospheric environment. **a** *n* = 1**. b** *n* = 2**. c** *n* = 3**.**

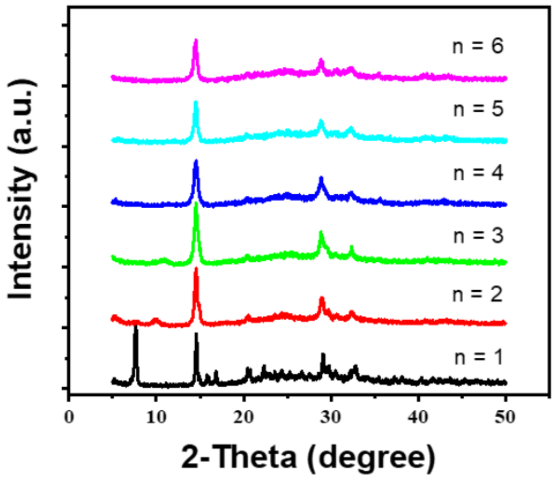

Supplementary Fig. 23 | XRD patterns. XRD patterns of the CDMA-based DJ perovskite films. It suggests that the $n > 1$ perovskite films have similar phase distributions.

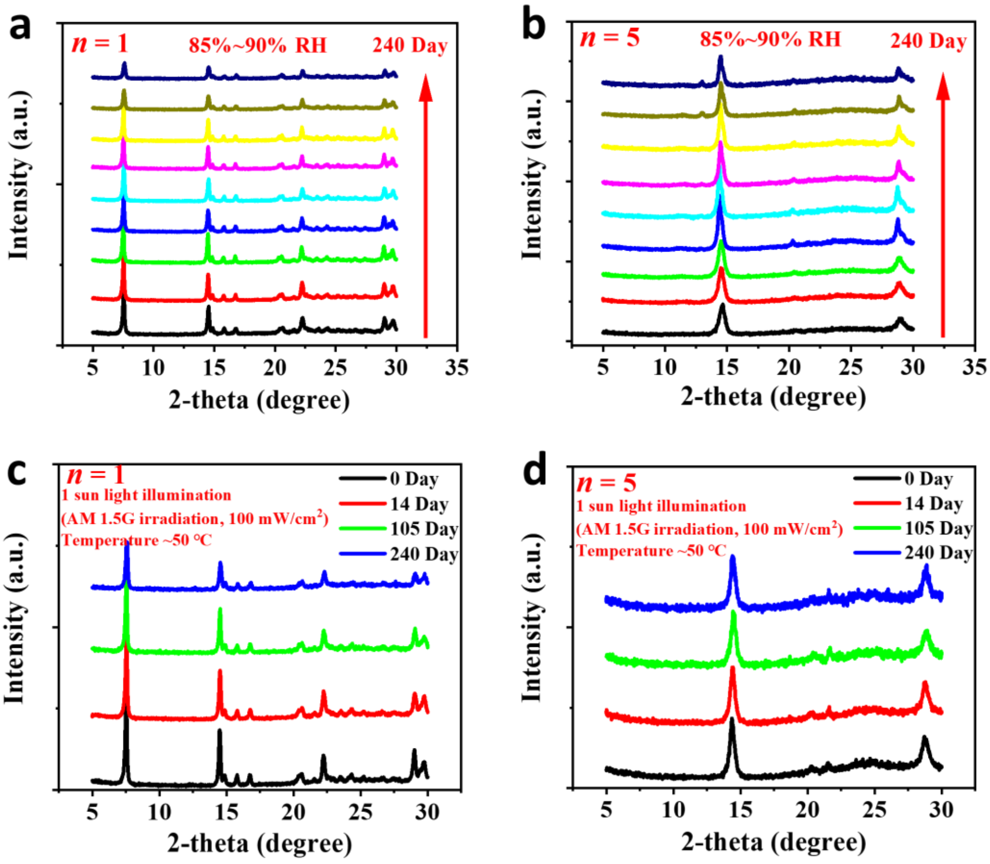

Supplementary Fig. 24 | Stability characterization. XRD patterns of the CDMA DJ perovskite films, showing an excellent film stability over 240 days aging by the moisture, thermal, light conditions. **a**, **b** $n = 1$ and $n = 5$ perovskite films for the humidity stability test in a constant temperature and humidity chamber, respectively. **c**, **d** $n = 1$ and $n = 5$ perovskite films for the light and thermal stability test in a glovebox with a 1-sun light and heating atmosphere, respectively.

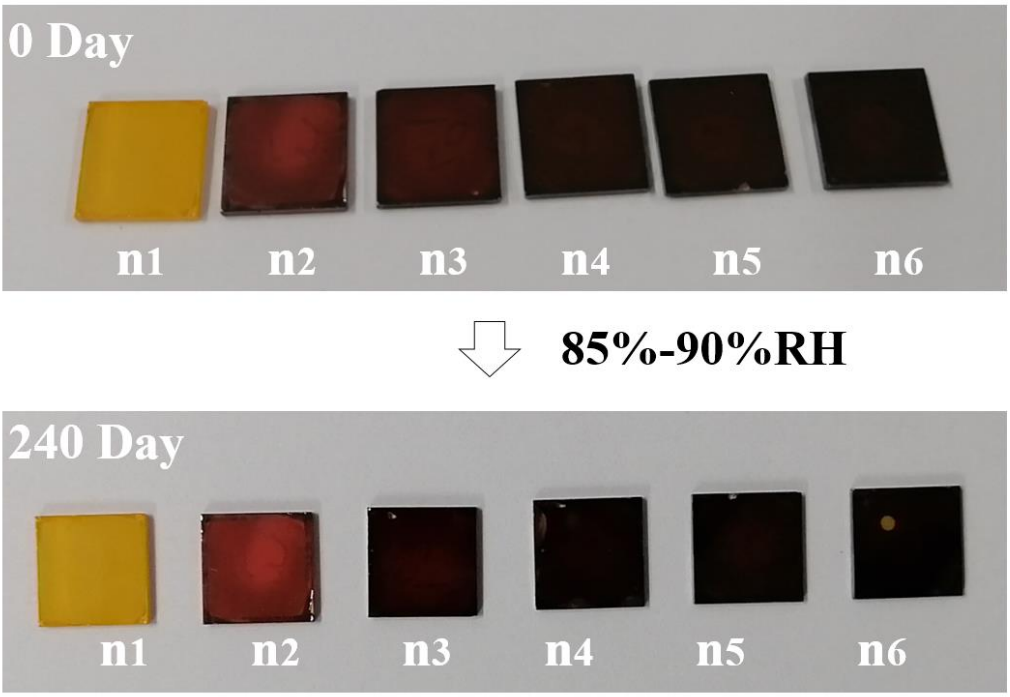

Supplementary Fig. 25 | Humidity stability characterization. Photographs of different *n*-value perovskite $(CDMA)(MA)_{n-1}Pb_nI_{3n+1}$ films under a constant temperature and humidity chamber.

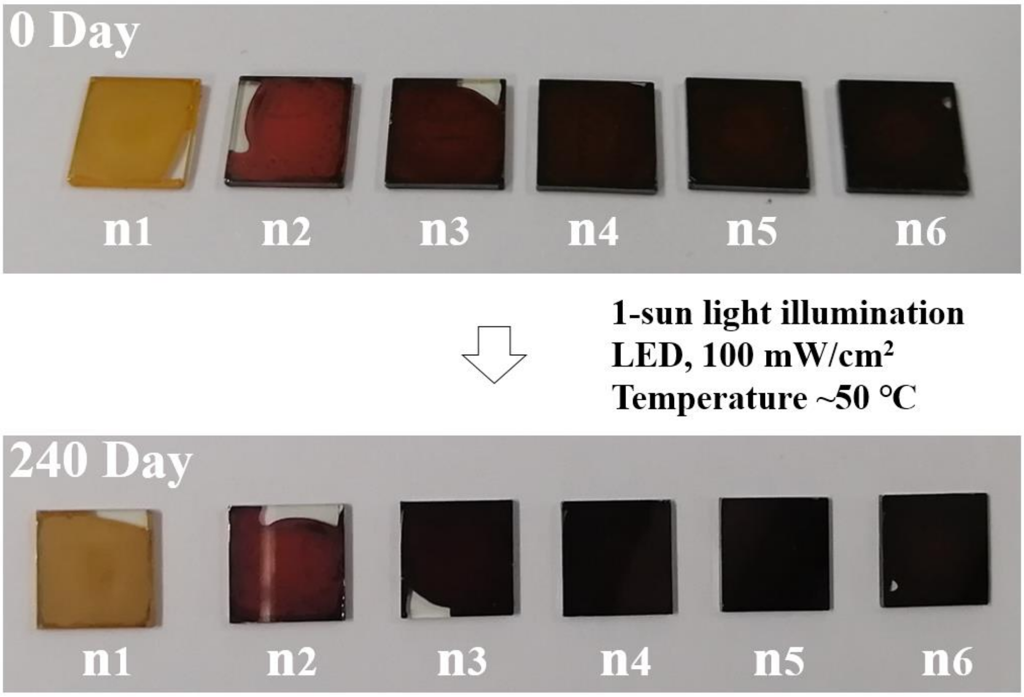

Supplementary Fig. 26 | Light stability characterization. Photographs of different n-value perovskite $(CDMA)(MA)_{n-1}Pb_{n}I_{3n+1}$ films for the light and thermal stability test in a glovebox with a 1-sun light and heating atmosphere, respectively.

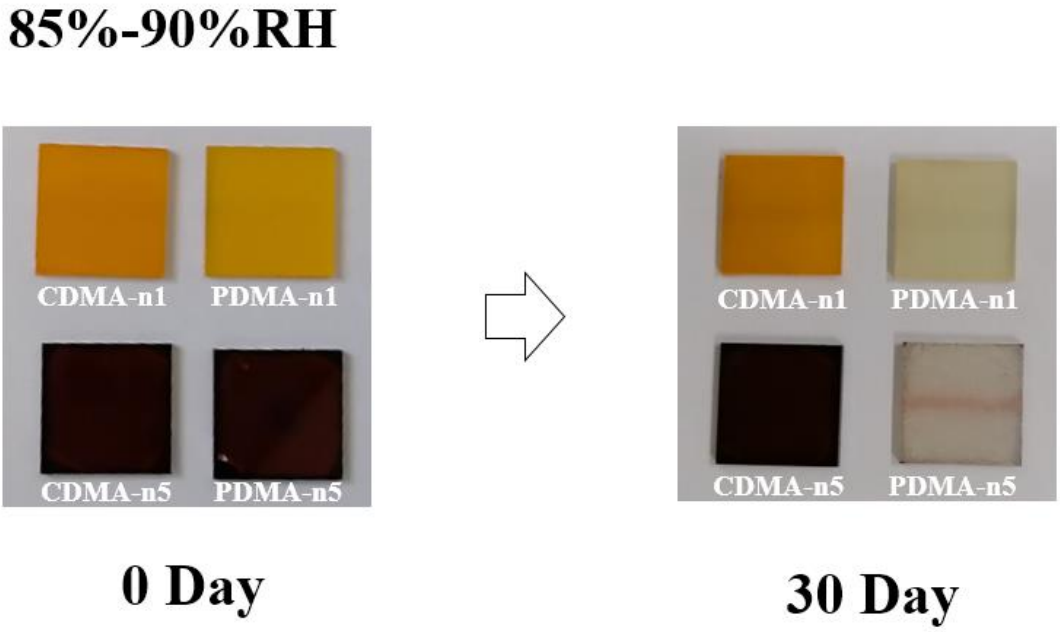

Supplementary Fig. 27 | Humidity stability characterization. Comparison of humidity stress of films based on the 0-displacement, rigid-cation and the slight interlayer-dislocation DJ perovskites. It indicates the design DJ perovskite series has excellent stability for scalable and stable photovoltaic applications.

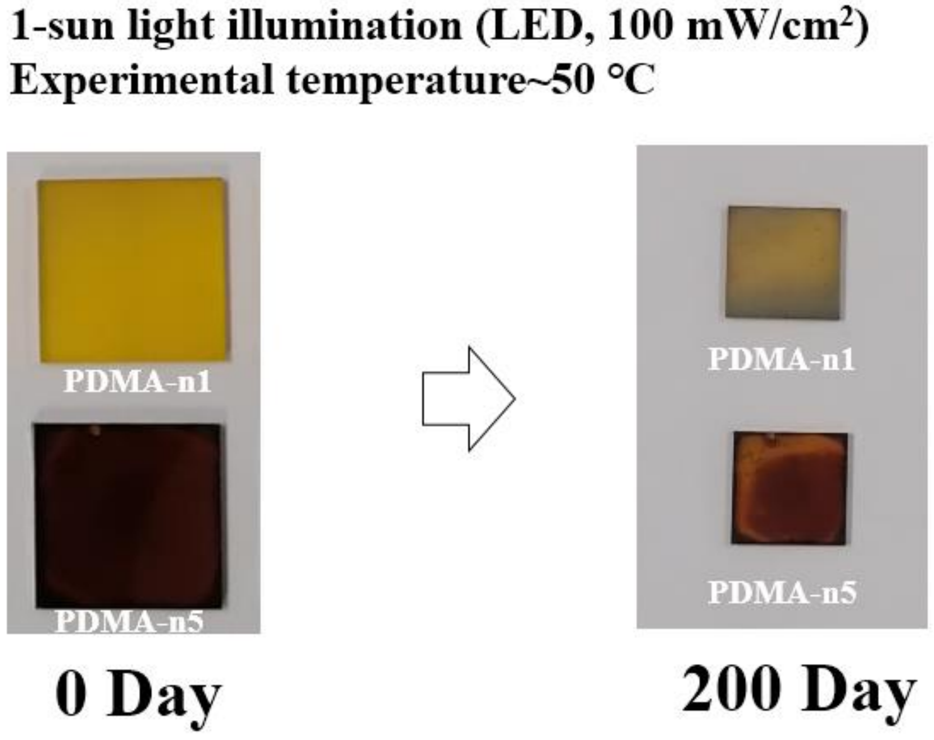

Supplementary Fig. 28 | Light stability characterization. Comparison of light and thermal stress of films based on the 0-displacement and rigid-cation DJ perovskites after the 200-day aging test. It indicates the 0-displacement and rigid-cation DJ perovskites have relatively poor stability compared to the DJ series.

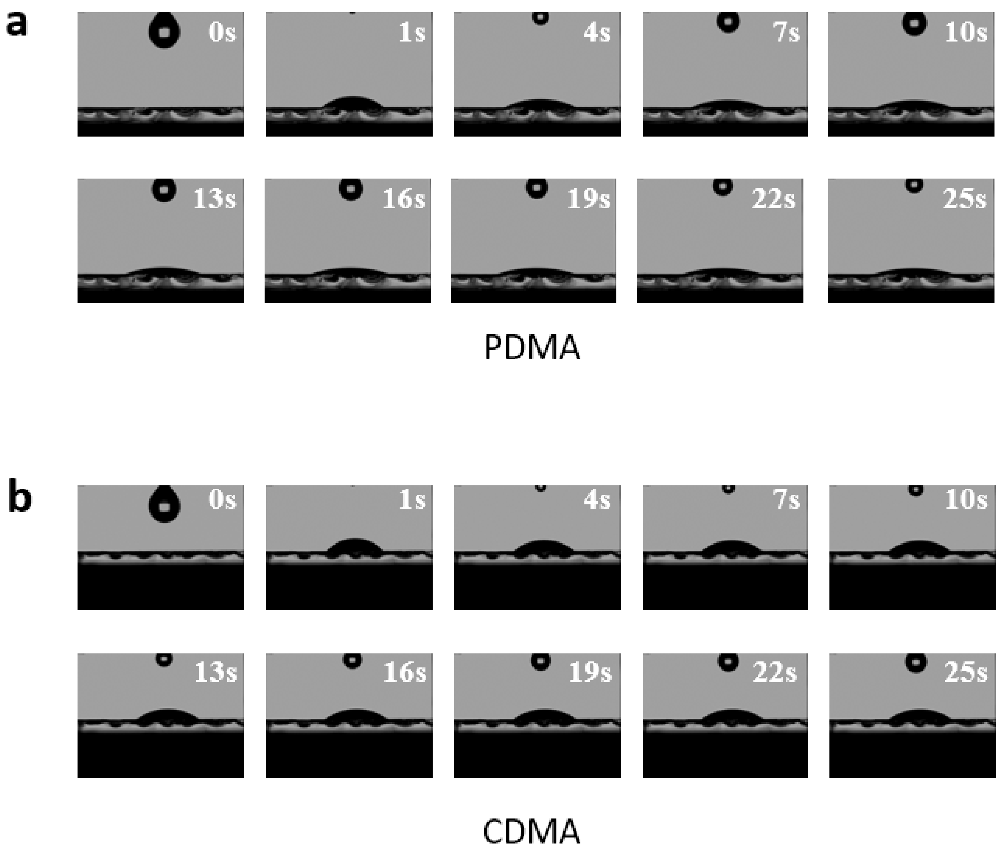

Supplementary Fig. 29 | Dynamic dissolving process of water contact angle characterization. a Photographs of time dependent contact angles of a water droplet on the surface of PDMA-based perovskite films. **b** Photographs of time dependent contact angles of a water droplet on the surface of CDMA-based perovskite films.

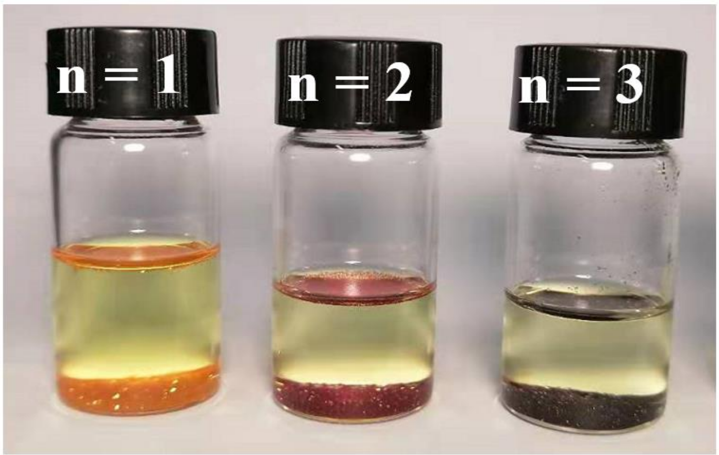

Supplementary Fig. 30 | Crystal growth of different perovskites. Photographs of different *n*-value $(CDMA)(MA)_{n-1}Pb_nI_{3n+1}$ (n = 1- 3) crystals in solution.

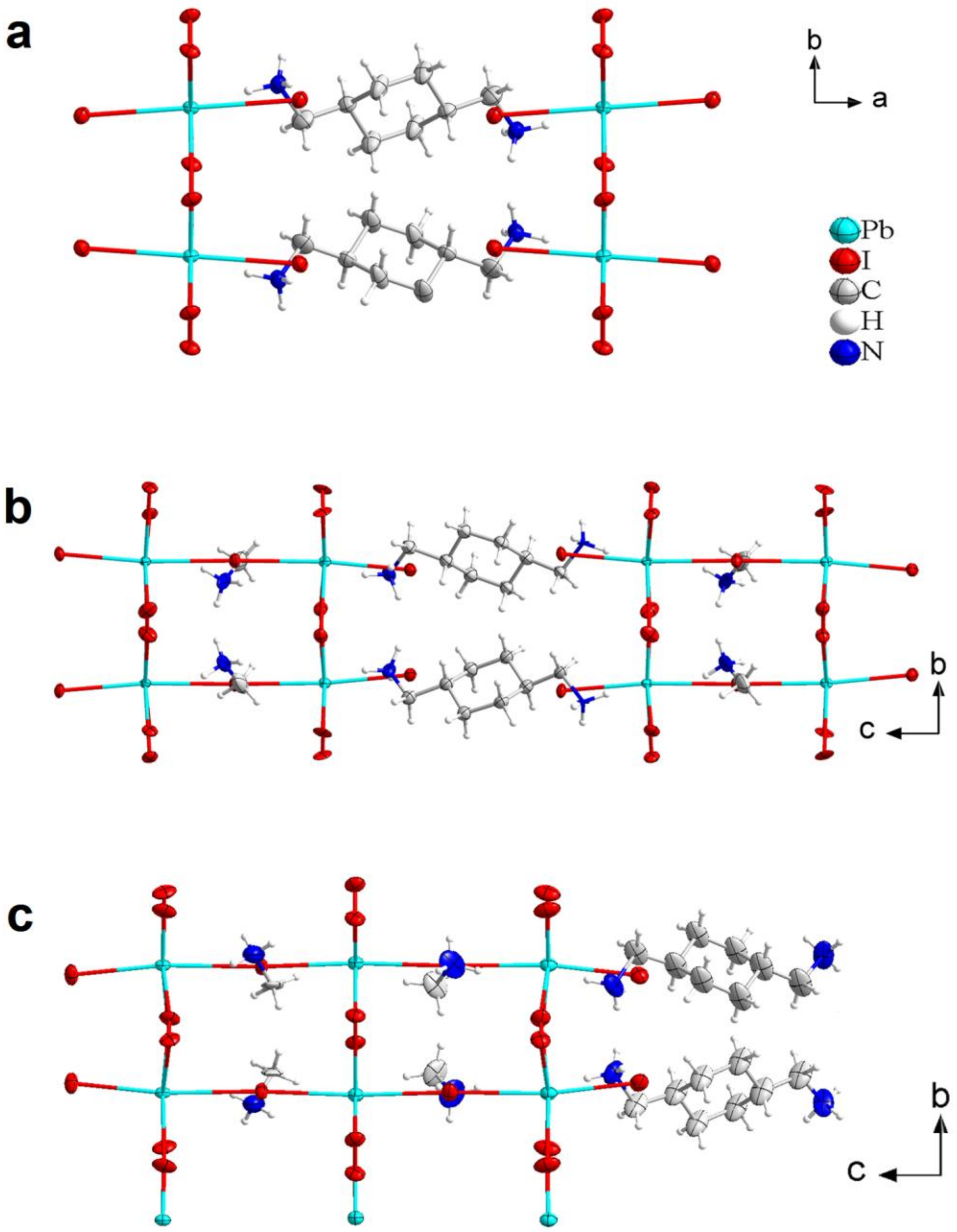

Supplementary Fig. 31 | ORTEP-style illustrations of the crystal structures. a Crystal structure of (CDMA)PbI_4 viewed down the c-axis. **b** Crystal structure of (CDMA)(MA)Pb_2I_7 viewed down the a-axis. **c** Crystal structure of (CDMA)$(MA)_2Pb_3I_{10}$ viewed down the a-axis.

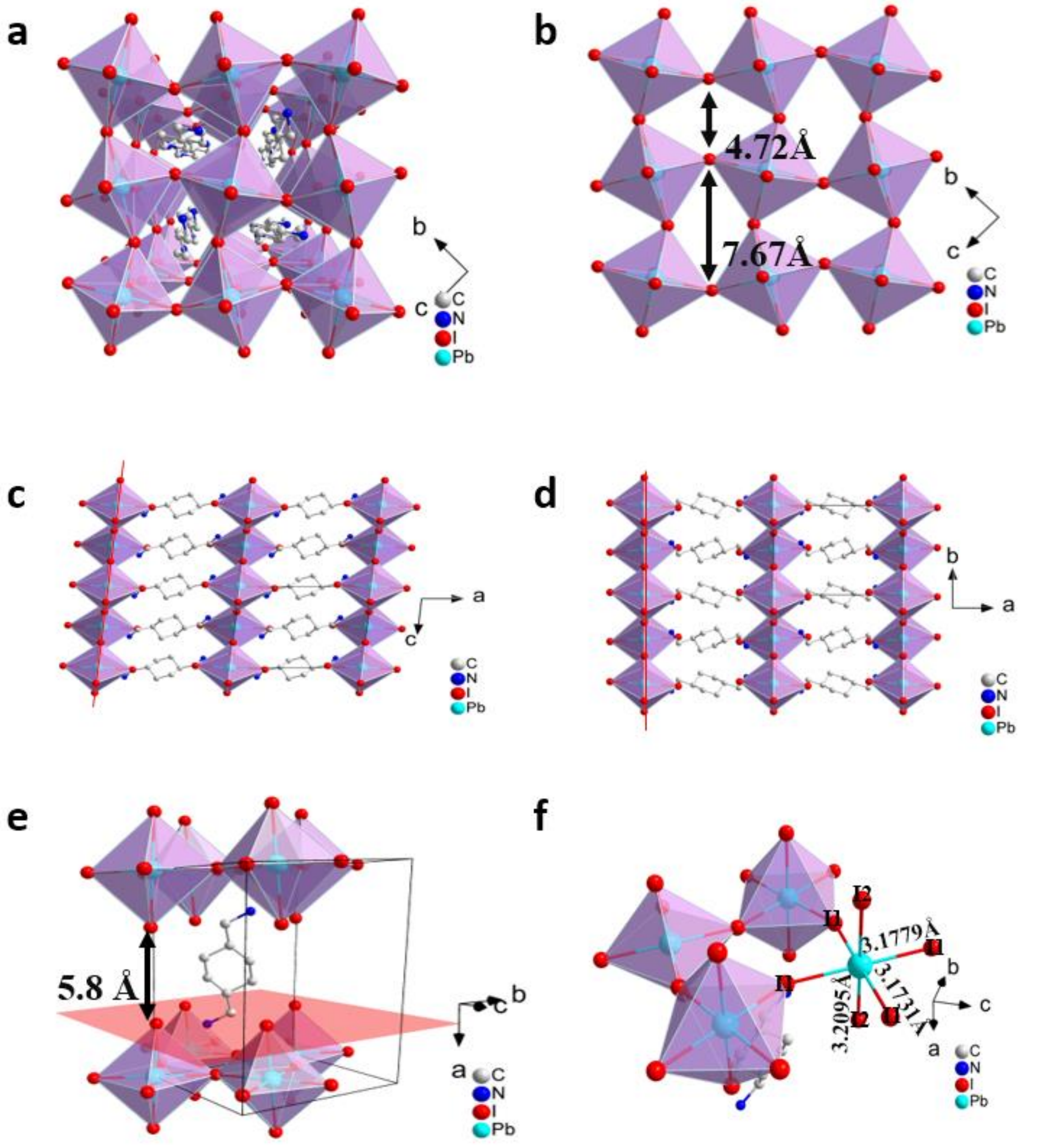

Supplementary Fig. 32 | Crystal structures of (CDMA)PbI_4. a Top-view of single-crystal structures. **b** Inorganic layer of the corner-sharing PbI_6 octahedra. Differing diagonal distances reveal a distorted square. **c, d** Side-view of single-crystal structures. **e** Layer distance of perovskite. **f** The PbI_6 octahedron and the corresponding bond length.

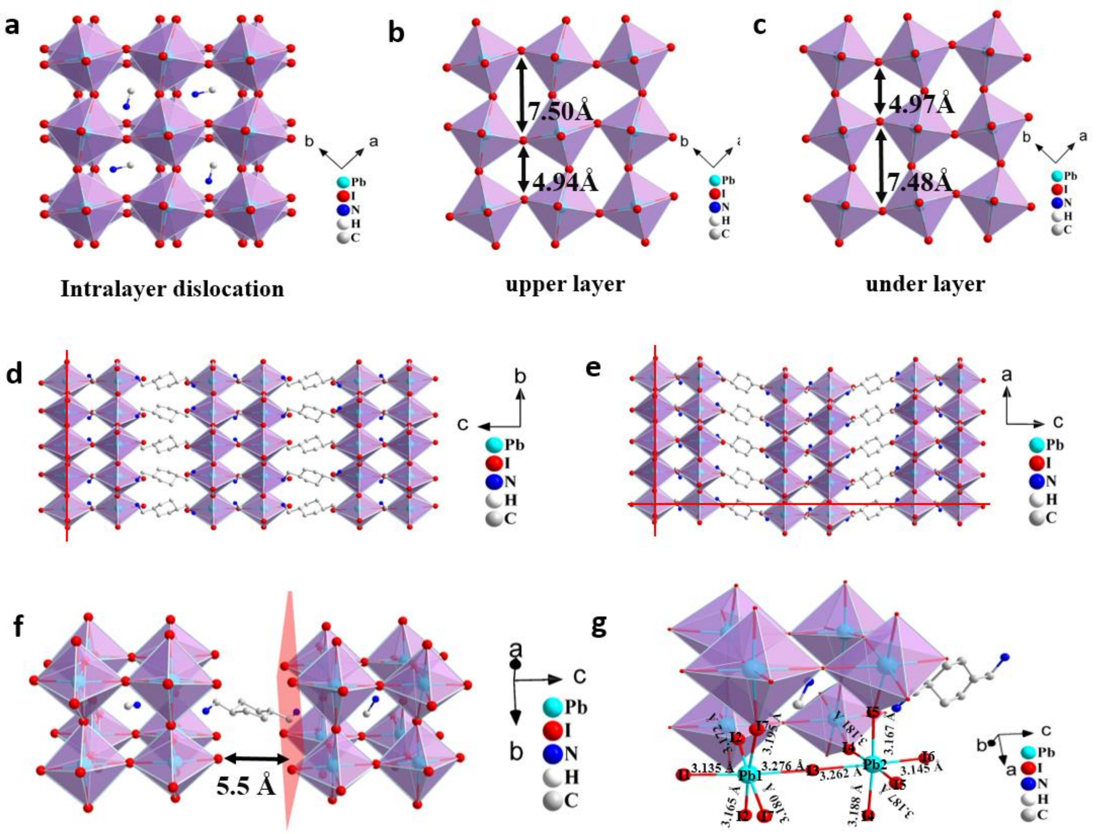

Supplementary Fig. 33 | Crystal structures of (CDMA)(MA)Pb_2I_7. **a** Top-view of single-crystal structures. **b, c** Inorganic layer of the corner-sharing PbI_6 octahedra. Differing diagonal distances reveal a distorted square. **d, e** Side-view of single-crystal structures. **f** Layer distance of perovskite. **g** The PbI_6 octahedron and the corresponding bond length.

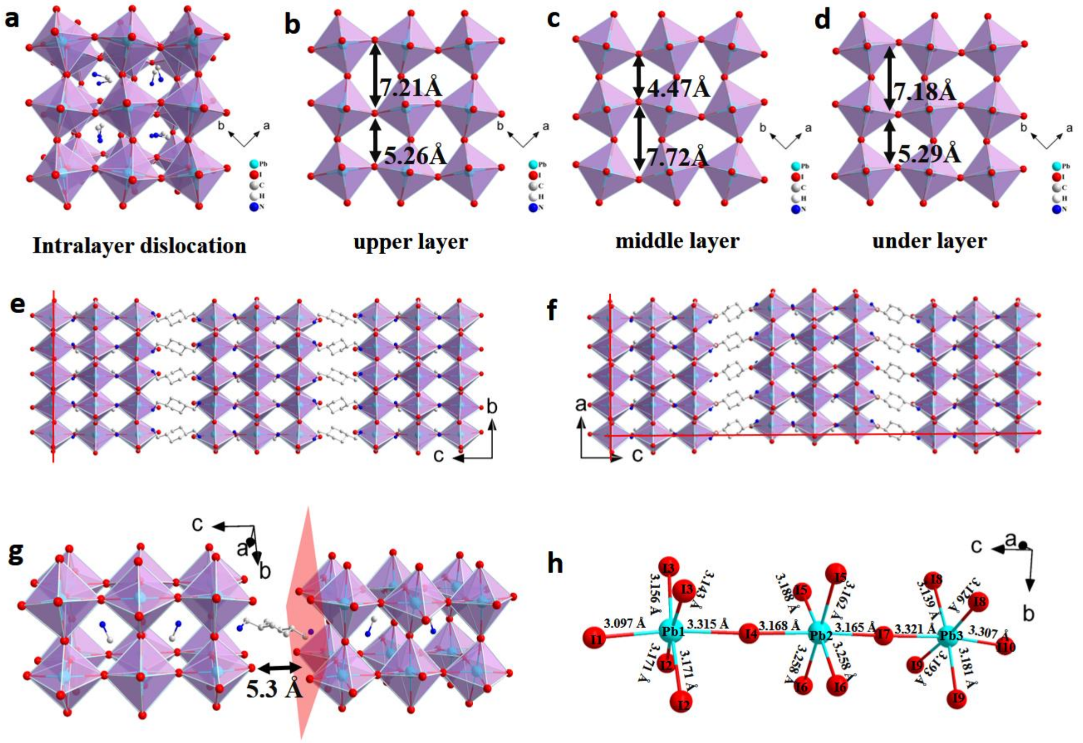

Supplementary Fig. 34 | Crystal structures of (CDMA)(MA)$_2$Pb$_3$I$_{10}$. a Top-view of single-crystal structures. **b, c, d** Inorganic layer of the corner-sharing PbI$_6$ octahedra. Differing diagonal distances reveal a distorted square. **e, f** Side-view of single-crystal structures. **g** Layer distance of perovskite. **h** The PbI$_6$ octahedron and the corresponding bond length.

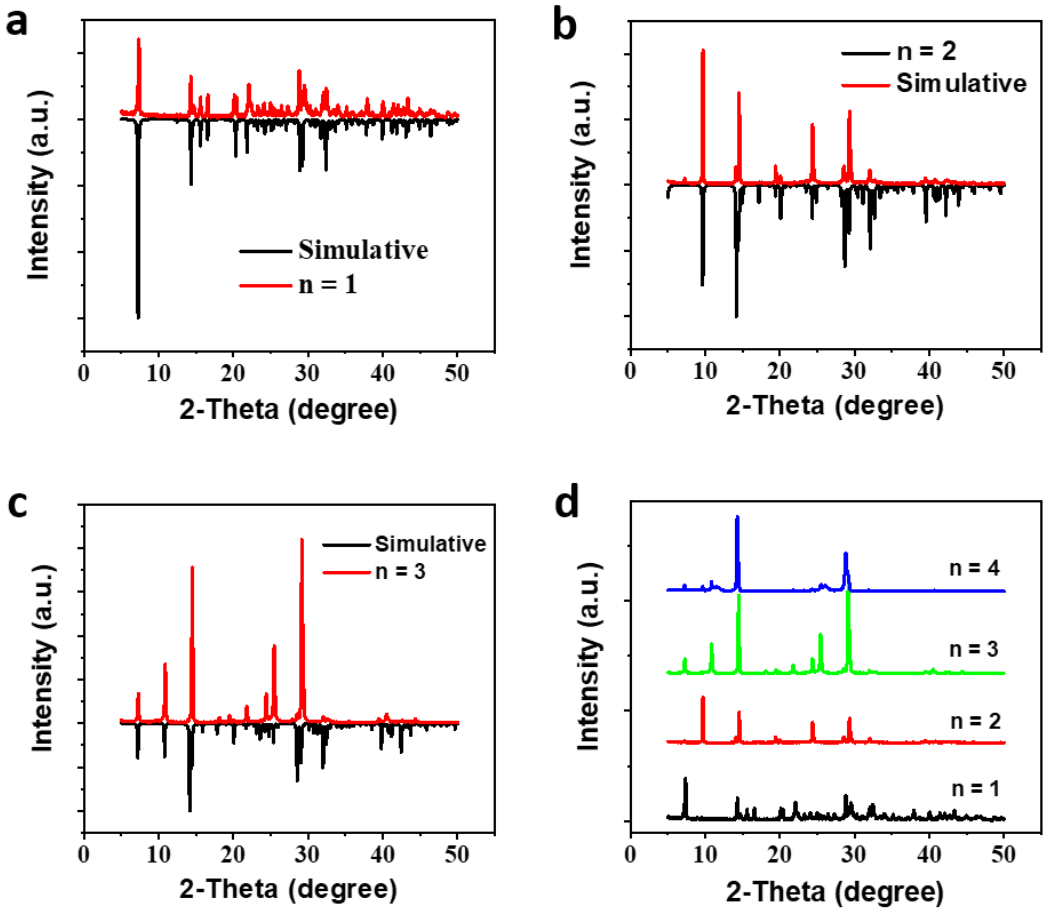

Supplementary Fig. 35 | XRD characterization. a, b, c XRD patterns of the CDMA DJ perovskites (*n* = 1-3), measuring by the corresponding pure crystal powder samples. **d** Comparison of XRD patterns of the CDMA DJ perovskites (*n* = 1-4), measuring by the corresponding pure crystal powder samples.

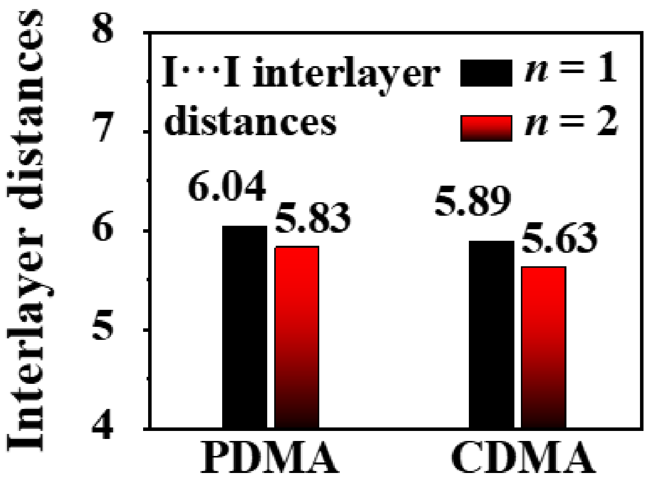

Supplementary Fig. 36 | Comparison of crystal structure parameters. The I···I distance between the inorganic layers of n =1 and n = 2 DJ perovskites.

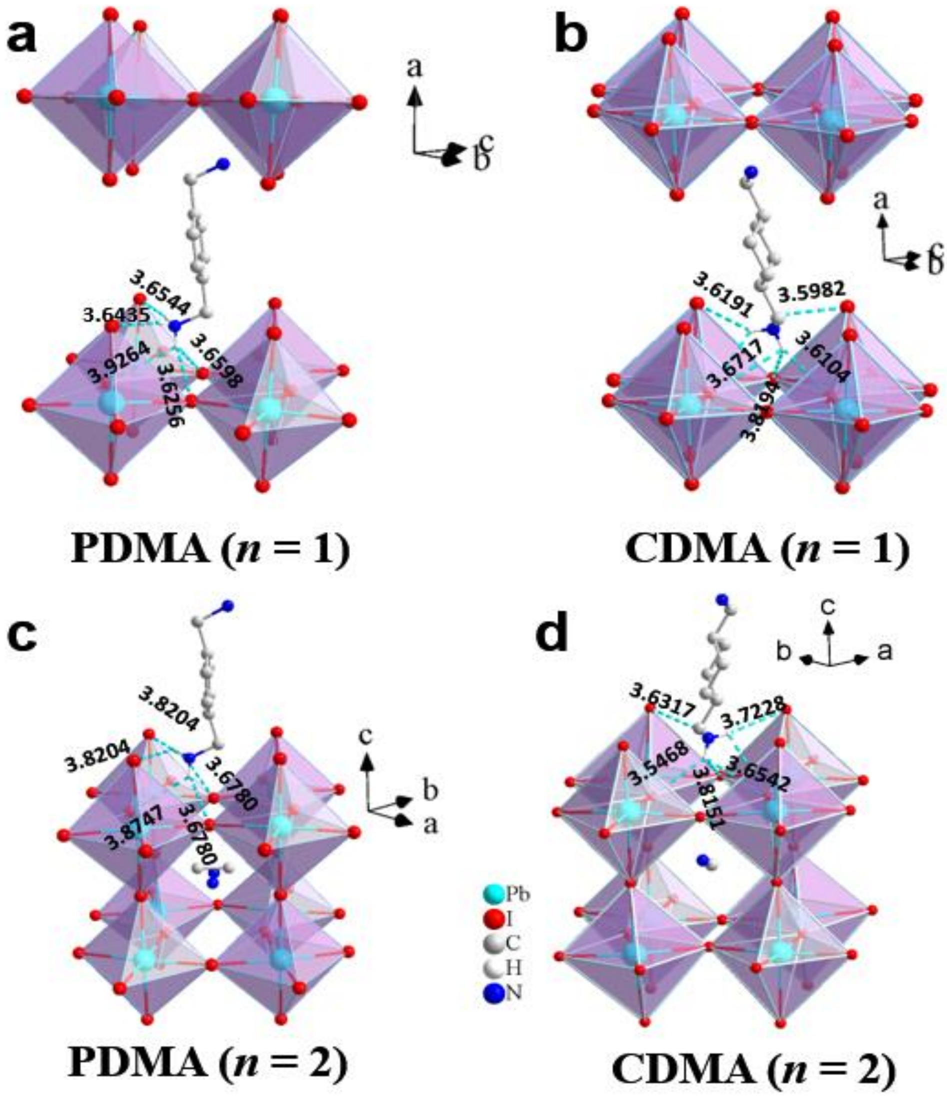

Supplementary Fig. 37 | Hydrogen bonds for PDMA and CDMA series from *n* = 1 and 2. a, c PDMA perovskite structures. **b, d** CDMA perovskite structures.

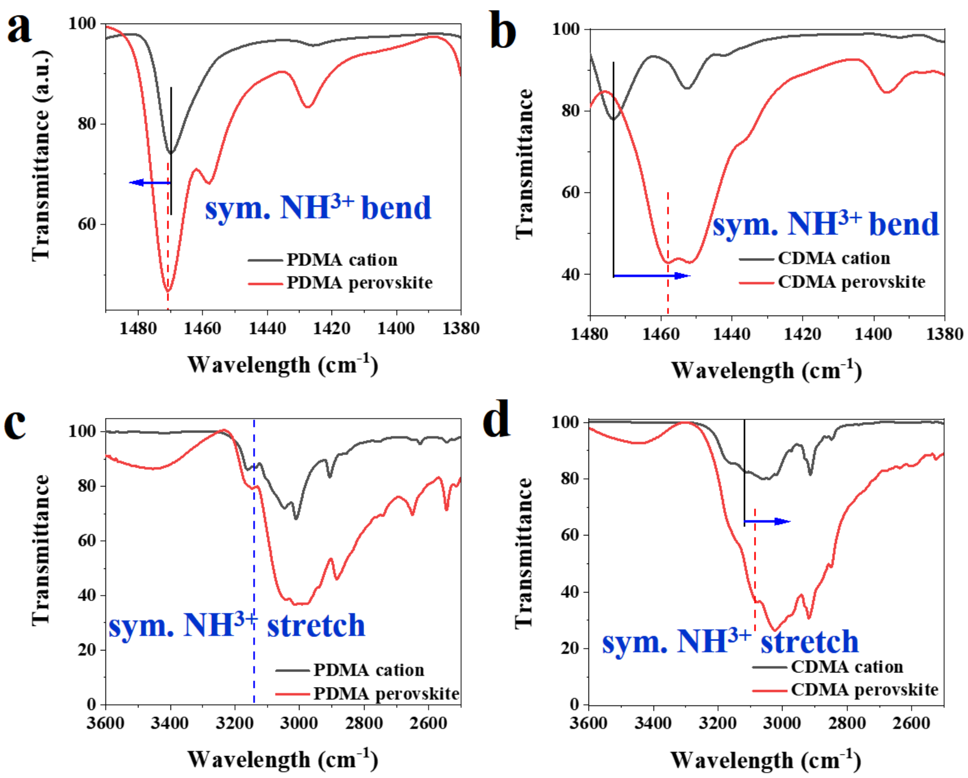

Supplementary Fig. 38 | FTIR spectra of the PDMA/CDMA cations and the corresponding *n* =1 CDMA- and PDMA-based DJ perovskites. a, c PDMA-based materials. **b, d** CDMA-based materials.

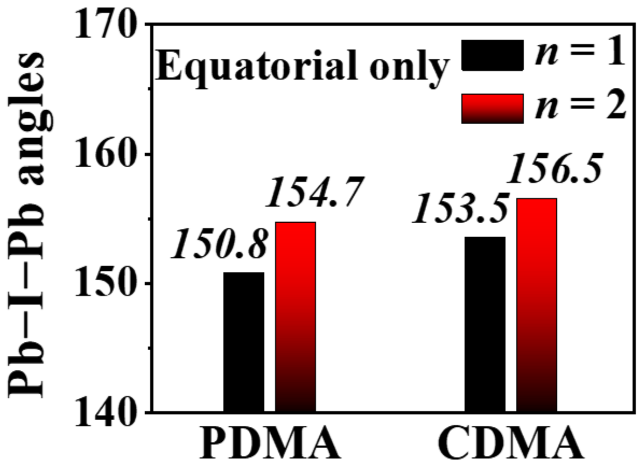

Supplementary Fig. 39 | Comparison of crystal structure parameters. Average equatorial Pb−I−Pb angles for PDMA and CDMA series from n = 1 and 2.

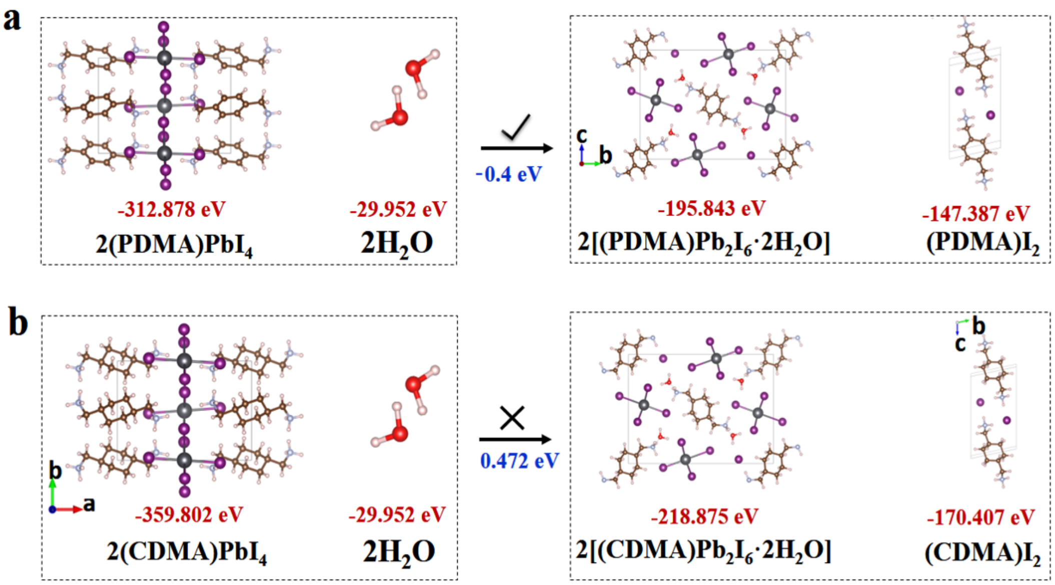

Supplementary Fig. 40 | DFT calculations. a DFT calculated formation energy of the PDMA-based materials. **b** DFT calculated formation energy of the CDMA-based materials.

Supplementary Tables

Supplementary Table 1. Summary of the best photovoltaic parameters of devices based on different *n*-value perovskites

**Devices**	**V_{OC} (V)**	**J_{SC} (mA cm^{-2})**	**FF (%)**	**PCE (%)**
$n = 1$	0.92	7.81	50.07	3.61
$n = 2$	1.04	8.58	54.84	4.92
$n = 3$	1.08	16.77	72.61	13.17
$n = 4$	1.08	16.62	76.14	13.71
$n = 5$	1.16	20.41	80.56	19.11
$n = 6$	1.07	18.69	76.09	15.30

Supplementary Table 2. Summary of the photovoltaic parameters of the recent reported 2D perovskite devices, fabricating via the spin-coating and blade-coating method.

NO.	Organic spacers in 2D perovskites	*n*	configuration	V_{OC} (V)	J_{SC} (mA cm^{-2})	FF(%)	PCE (%)	REF
**Spin-coating devices**								
1	2,3,5,6-tetrafluoro-1,4-benzenedimethanammonium	DJ-4	ITO/PEDOT:PSS/DJ perovskite/PCBM/BCP/Ag	1.10	19.58	77.20	16.62	4
2	PhDMA	DJ-4	ITO/PEDOT:PSS/DJ perovskite/PCBM/BCP/Ag	1.03	14.63	66.97	10.11	4
3	propanediammonium	DJ-4	FTO/TiO_2/perovskite/Spiro-OMeTAD/Au	0.98	18.86	64	12.17	5
4	1,4-phenylenedimethanammonium	DJ-4	FTO/c-TiO_2/perovskite/Spiro-OMeTAD/Au	1.15	21.10	62.58	15.09	6
5	thieno[3,2-b]thiophene-2,5-diyldimethanaminium	DJ-4	ITO/PEDOT:PSS/perovskite/PCBM/BCP/Ag	1.03	22.38	81.64	18.82	7
6	3-(aminomethyl)piperidinium	DJ-4	ITO/(NiO_x/PTAA)/perovskite/$PC_{61}BM$/BCP/Ag	1.24	19.51	77.27	18.67	8
7	1,3-propanediammonium	DJ-4	FTO/TiO_2/ perovskite/Spiro-OMeTAD/Au	1.03	20.20	71	14.74	9
8	trans-1,4-cyclohexanediamine	DJ-4	FTO/SnO_2/perovskite/Spiro-MeOTAD/Au	1.064	21.03	67.1	15.01	10
9	3-(aminomethyl)piperidinium	DJ-4	FTO/PEDOT:PSS/perovskite/C60/BCP/Ag	1.09	13.69	81.04	12.04	11
10	propane-1,3-diammonium	DJ-4	ITO/SnO_2/perovskite/Spiro-OMeTAD/Ag	1.09	18.9	77.7	16.0	12
11	1,5-naphthalene diammonium	DJ-4	FTO/c-TiO_2/SnO_2/perovskite/spiro-OMeTAD/Au	1.05	20.45	69.74	15.08	13
12	propane-1,3-diammonium	DJ-4	ITO/SnO_2/perovskite/Spiro-OMeTAD/Ag	1.10	17.30	72.5	13.8	14
13	2,5-thiophenedimethylammonium	DJ-5	ITO/PEDOT:PSS/perovskite/PCBM/BCP/Ag	1.07	19.55	75.46	15.75	15
14	1,4-butanediammonium	DJ-5	ITO/PTAA/perovskite/PCBM/BCP/Ag	1.11	16.07	81.45	14.53	16
15	1,4-butanediammonium	DJ-5	ITO/SnO_2/ perovskite/spiro-OMeTAD/Au	1.064	19.71	76.6	16.07	17
16	1,4-butanediammonium	DJ-5	ITO/PEDOT:PSS/perovskites/PCBM/LiF/Al	1.04	20.01	78.64	16.38	18
17	1,4-phenylenedimethanammonium	DJ-5	ITO/PEDOT:PSS/perovskite/PC61BM/BCP/Ag	1.08	20.90	48	10.86	19
18	thieno[3,2-b]thiophene-2,5-diyldimethanaminium	DJ-5	ITO/PEDOT:PSS/perovskite/PC61BM/BCP/Ag	1.08	21.35	86.16	19.87	20
19	1,4-butanediammonium	DJ-6	FTO/SnO_2/perovskite/Spiro-OMeTAD/Au	1.11	21.22	73	17.17	21
20	m-phenylenediammonium	DJ-6	FTO/TiO_2/perovskite/spiro-OMeTAD/Au	0.82	14.74	51	6.16	22
21	2,3,5,6-tetrafluoro-1,4-benzenedimethanammonium	DJ-10	ITO/SnO_2/perovskite/Spir-o-OMeTAD/Au	1.05	18.76	77.31	15.24	23
Blade-coated devices								
22	butylammonium	RP-5	ITO/PEDOT:PSS/<n>=5/PCBM/BCP/Cu	1.05	18.35	74.93	14.4	24
24	butylammonium	RP-4	ITO/PTAA/perovskite/C_{60}/BCP/Ag	1.15	18.15	74.17	15.47	25
25	butylammonium	RP-4	ITO/NiO/perovskite/PCBM/BCP/Au	1.22	18.68	71.6	16.36	26
26	1,4-phenylenedimethanammonium	DJ-5	ITO/PTAA/perovskite/C_{60}/BCP/Ag	1.06	18.32	76.46	14.87	This work
27	1,4-cyclohexanedimethanammonium	DJ-5	ITO/PTAA/perovskite/C_{60}/BCP/Ag	1.16	20.41	80.56	19.11	This work

Note: Purple fonts represent blade-coated devices. DJ-5 represents the nominal *n* = 5 DJ perovskite solar cells. RP-5 represents the nominal *n* = 5 RP perovskite solar cells.

Supplementary Table 3. Comparison of lead-based solar cell stability with the representative literature works. E represent encapsulated cells.

**Perovskites**	**Type**	**PCE**	**RH** (Proportion of the initial efficiency/ Humidity /Time)	**Thermal** (Proportion of the initial efficiency/ Temperature /Time)	**Operation** (Proportion of the initial efficiency/Time)	**Ref.**
$isoBA_2(Cs_{0.02}MA_{0.64}FA_{0.34})_4Pb_5I_{16}$	RP	16%	**75%**/85% RH/144 h	-	-	27
$GA(MA)_5Pb_5I_{16}$	CAI	22.26%	E/**93.81%**/25% RH/1200 h	**56.26%**/60 h	-	28
$(NpMA)_2(MA)_3Pb_4I_{13}$	RP	17.25%	-`	**80%**/80℃/600 h	-	29
$(AnMA)_2(MA)_3Pb_4I_{13}$	RP	14.47	-	**77%**/80℃/600 h	-	29
$(ThMA)_2FA_4Pb_5I_{16}$	RP	19.06%	**99%**/30% RH/552 h	**96%**/80℃/576 h	-	30
$BA_2MA_3Pb_4I_{13}$	RP	-	E/**40%**/85% RH/1000 h	-	-	31
$(F\text{-}PEA)_2(FA)_4Pb_5I_{16}$	RP	21.07%	E/**90%**/85% RH/1000 h	**97%**/85℃/1500 h	-	32
$(F\text{-}PEA)_2(MA)_4Pb_5I_{16}$	RP	17.05%	-	**78%**/85℃/1500 h	-	32
$(4FPEA)_2(MA)_4Pb_5I_{16}$	RP	17.3%	**93%**/55–65% RH/500 h	**100%**/55℃/500 h	-	33
$(4FPEA)_2(MA)_4Pb_5I_{16}$	RP	11.10%	**70%**/55–65% RH/500 h	**65%**/55℃/500 h	-	33
$(PEA)_2(MA)_3Pb_4I_{13}$	RP	18.5%	**90%**/40% RH/1200 h	-	**95%**/500h	34
$(PEA)_2(MA)_4Pb_5I_{16}$	RP	13.5%	**70%**/40–50% RH/960 h	-	-	35
$(F\text{-}PEA)_2(MA)_4Pb_5I_{16}$	RP	14.5%	**90%**/40–50% RH/960 h	-	-	35
$(MeO\text{-}PEAI)_2(MA)_4Pb_5I_{16}$	RP	9.9%	**97%**/40–50% RH/960 h	-	-	35
$GA_{0.2}BA_{1.8}MA_5Pb_6I_{19}$	RP	18.24%	**90%**/50 ± 5% RH/1340 h	-	-	36
$(F\text{-}PEA)_2MA_3Pb_4I_{12}$	RP	18.10%	**90%**/40–50% RH/720 h	**80%**/80℃/720h	-	37
$(0.1PEA\text{-}BA)_2(MA)_4Pb_5I_{16}$	RP	14.09%	**75%**/55 ± 5% RH/1000 h	-	-	38
$iBA_2(FA\text{-}MA)_{17}Pb_{18}I_{55}$	RP	20.12%	**77%**/85% RH/1000 h	**88%**/85℃/360 h	**96%**/360h	39
$PMA_2FA_{58}Pb_{59}I_{178}$	RP	19.33%	**63%**/40–50% RH/120 h	-	-	40
$(PEA)_2(MA)_3Pb_4I_{13}$	RP	17.03%	**90%**/40% RH/960 h	**90%**/85℃/1600 h	-	41
Cs-doped $(BA)_2(MA)_3Pb_4I_{13}$	RP	13.7%	**81%**/85% RH/24 h	**85%**/85℃/16 h		42
$(BA)_2(MA)_3Pb_4I_{13}$	RP	12.3%	**45%**/85% RH/24 h	**55%**/85℃/16 h		42

$(PPA)_2(Cs_{0.05}(FA_{0.88}MA_{0.12})_{0.95})_3Pb_4(I_{0.88}Br_{0.12})_{13}$	RP	14.76%	**96%**/50% RH/200 h	-	-	[43]
$(BA)_2(MA,FA)_3Pb_4I_{13}$	RP	12.81%	**88%**/40–60% RH/1300 h	-	-	[44]
$PEA_2MA_4Pb_5I_{16}$	RP	18.04%	-	-	**95.2%**/10h	[45]
$(BA)_2(MA)_3Pb_4I_{13}$	RP	13.2%	-	-	E/**95%**/500h	[24]
$(BA)_2(MA)_3Pb_4I_{13}$	RP	12.51%			**0%**/60h	[46]
$(BA)_2(MA)_3Pb_4I_{13}$	RP	16.25%	**93.8%**/65±10%RH/4680 h	**95.7%**/85℃/558 h	-	[47]
$(MTEA)_2(MA)_4Pb_5I_{16}$	RP	18.06%	-	-	**87.1%**/1000h	[48]
$BA_2MA_3Pb_4I_{13}$	RP	17.74	**90%**/40–50% RH/2400 h	**93%**/85℃/2400 h	-	[49]
$(BDA)FA_4Pb_5I_{16}$	DJ	16.07%	**94%**/15–20% RH/1080 h	**86%**/60℃/360 h	-	[17]
$(PDA_{0.9}PA_{0.2})(FA)_3Pb_4I_{13}$	DJ	16.0%	-	**90%**/85℃/800 h	-	[12]
$(BEA)_{0.5}MA_3Pb_3I_{10}$	DJ	14.86%	**80%**/85% RH/300 h	**80%**/85℃/100 h	-	[50]
$(TTDMA)MA_3Pb_4I_{13}$	DJ	18.82%	**98%**/30 ± 5% RH/960 h	**94%**/80℃/740 h	-	[7]
$(3AMP)(MA_{0.75}FA_{0.25})_3Pb_4I_{13}$	DJ	12.04%	**22%**/50–70% RH/47.5 h	-	-	[11]
$(PDMA)MA_3Pb_4I_{13}$	DJ	15.81%	**89%**/30% RH/744 h	-	-	[6]
$(PDA)(FA)_3Pb_4I_{13}$	DJ	13.8%	-	**85%**/85℃/540 h	-	[14]
$(ThDMA)MA_4Pb_5I_{16}$	DJ	15.75%	-	**90%**/80℃/360 h	-	[15]
$(3AMP)(MA_{0.75}FA_{0.25})_3Pb_4I_{13}$	DJ	18.67%	**90%**/45±5% RH/1440 h	**90%**/85℃/480 h	-	[8]
$(4F\text{-}PhDMA)MA_3Pb_4I_{13}$	DJ	16.62%	-	**94%**/80℃/360 h	-	[4]
$(BDA)(Cs_{0.1}FA_{0.9})_4Pb_5I_{16}$	DJ	18.2%	**100%**/80% RH/800 h	**90%**/85℃/800 h	E /**95%**/800h	[51]
$(CHDA)MA_3Pb_4I_{13}$	DJ	15.01%	**94.7%**/50–60% RH/1248 h	**74.4%**/70℃/68 h	**80.7%**/4.5h	[10]
$(BDA)(MA)_4Pb_5I_{16}$	DJ	14.53%	**85%**/50 ± 5% RH/900 h	-	-	[16]
$(3AMP)(MA_{0.75}FA_{0.25})_3Pb_4I_{13}$	DJ	16.25%	**80%**/45 ± 5% RH/840 h	-	-	[52]
$(PDA)MA_3Pb_4I_{13}$	DJ	13.0%	E/**90%**/85% RH/1000 h	-	-	[31]
$(FA_{0.98}MA_{0.02})_{0.95}Pb(I_{0.98}Br_{0.02})_3$	3D	25.0%	E-**95%**/85% RH-85℃/1000 h		**98%**/1500h	[53]

$(FAPbI_3)_{1-0.03}(MDA{:}Cs)_{0.03}$	3D	24.4%	**80%**/15-25% RH-85℃/1300 h		**90%**/400 h	[54]
$FAPbI_3$@MAFa	3D	24.1%	-	**90%**/85℃/500 h	**90%**/500 h	[55]
$FAPbI_3$:0.38$MDACl_2$	3D	25.8%	-	-	**90%**/500 h	[56]
$FAPbI_3$-$HCOO^-$	3D	25.6%	**90%**/20% RH/1000 h	**80%**/60℃/1000 h	**85%**/450 h	[57]
$(FA_{0.83}MA_{0.17})_{0.95}Cs_{0.05}Pb(I_{0.9}Br_{0.1})_3$	3D	19.8%	**86%**/40–50% RH/100 h	E-**80%**/70-75℃/1072h	-	[58]
$(FAPbI_3)_{0.95}(MAPbBr_3)_{0.05}$	3D	23.3%	**80%**/85% RH/1008 h	-	**95%**/1,370 h	[59]

Supplementary Table 4. Decay lifetime of the perovskite films.

Sample	$\tau_{1\ (ns)}$/ratio	$\tau_{2\ (ns)}$/ratio
**PDMA**	7.21/32.39%	51.63/67.61
**CDMA**	4.79/47.18%	27.8/52.82%

The time-resolved photoluminescence decay curves can be fitted by the bi-exponential function:

$$y = A_1\exp(-t/\tau_1) + A_2\exp(-t/\tau_2) + y_0$$

Where A_1 and A_2 are the relative amplitudes; and τ_1 and τ_2 are the decay time constants.[60,61]

Supplementary Table 5. Fitting decay components for perovskite films

Perovskite	Phases	A1	τ_1/ps	A2	τ_2 /ps
**PDMA**	$n = 2$	10.17	0.24	0.09	0.003
	$n = 3$	0.42	0.53	0.26	14.07
	$n = 4$	0.23	1.86	0.29	36.09
	$n = 5$	0.19	4.52	0.29	77.44
**CDMA**	$n = 3$	1.33	0.21	0.24	6.58
	$n = 4$	0.37	0.42	0.30	49.67
	$n = 5$	0.20	0.53	0.37	163.62
	$n = 6$	0.07	1.86	0.39	528

The kinetics extracted from TA spectra are fitted with multi-exponential decay function:

$$I(t) = 1/2\sum_{i=1}^{N} H_i\ (t)A_i\exp(\frac{-t}{\tau_i})$$

where t is the probe time delay, $H_i(t)$ = [1+erf(-t/r-r/2τ_i)] is the rising function, r (~ 0.1 ps) is the Gaussian laser pulse width, A_i is the amplitude or pre-exponential function, and τ_i is the decay time. The fast component (τ_1) and slow component (τ_2) can be assigned to energy transfer and charge transfer process, respectively.[62]

Supplementary Table 6. Crystal data and structure refinement for perovskite $(CDMA)(MA)_2Pb_3I_{10}$.

**Parameters**	**Perovskite**
CCDC number	2357881
Empirical formula	$(CDMA)(MA)_2Pb_3I_{10}$
Formula weight	2098.96
Temperature/*K*	273.15
Crystal system	orthorhombic
Space group	$Pna2_1$
a/Å	8.8042(15)
b/Å	8.8303(16)
c/Å	48.841(8)
α/°	90
β/°	90
γ/°	90
Volume/Å3	3797.1(11)
Z	4
ρ_{calc} g/cm^3	3.672
μ mm^{-1}	21.407
F(000)	3584
Radiation	MoKα (λ = 0.71073)
2*Θ* range for data collection/°	4.688 to 49.996
Index ranges	$-10 \leq h \leq 10$, $-7 \leq k \leq 10$, $-57 \leq l \leq 58$
Reflections collected	18973
Independent reflections	6615 [R_{int} = 0.0944, R_{sigma} = 0.1175]
Data/restraints/parameters	6615/157/250
Goodness-of-fit on *F*2	1.031
Final *R* indexes [I>=2σ (I)]	R_1 = 0.0835, wR_2 = 0.2264
Final *R* indexes [all data]	R_1 = 0.1138, wR_2 = 0.2491
Largest diff. peak/hole / e Å^{-3}	3.70/-2.65
Flack parameter	0.42(4)

Supplementary Table 7. Fractional Atomic Coordinates ($\times 10^4$) and Equivalent Isotropic Displacement Parameters ($Å^2 \times 10^3$) for perovskite (CDMA)$(MA)_2Pb_3I_{10}$. U_{eq} is defined as 1/3 of the trace of the orthogonalised U_{IJ} tensor.

Atom	*x*	*y*	*z*	*U*(eq)
Pb(1)	5749(2)	5002(3)	6528.1(4)	18.7(6)
Pb(2)	5668.3(16)	5086.6(16)	5201.5(8)	25.0(4)
Pb(3)	5829(2)	5018(3)	3874.4(5)	29.0(8)
I(2)	8672(4)	2919(6)	6479.6(11)	34.3(13)
I(4)	5785(5)	4949(6)	5849.4(10)	32.0(11)
I(3)	2891(4)	7133(6)	6518.0(13)	41.9(13)
I(1)	5525(5)	4627(7)	7157.3(9)	37.6(12)
I(5)	3838(3)	8168(3)	5205.8(16)	36.5(7)
I(6)	2648(3)	3030(3)	5202.6(16)	34.6(7)
I(10)	5641(5)	4603(6)	3250.8(10)	39.4(12)
I(7)	5825(5)	4982(6)	4554.3(11)	38.9(14)
I(9)	3774(5)	2070(6)	3930.2(12)	40.5(14)
I(8)	8011(5)	7830(7)	3883.8(14)	51.6(16)
C(1)	9950(80)	4200(70)	5773(15)	46(14)
N(1)	10760(70)	5430(70)	5902(13)	60(14)
C(3)	5800(60)	9510(60)	3272(8)	61(9)
N(3)	6520(60)	10790(60)	3427(11)	51(11)
C(2)	4910(60)	10780(100)	4654(16)	63(19)
C(7)	6590(50)	10570(70)	2820(8)	67(8)
C(5)	4360(50)	8800(60)	2850(8)	66(8)
C(8)	6000(60)	11230(70)	2556(9)	69(8)
C(6)	3770(50)	9430(70)	2584(9)	67(8)
C(4)	5280(50)	10010(60)	2998(8)	66(8)
C(9)	5050(50)	10050(60)	2407(8)	70(8)
C(10)	4450(60)	10680(60)	2143(9)	70(9)
N(4)	3520(50)	9600(70)	1982(9)	59(12)
N(2)	6230(60)	9960(70)	4537(14)	71(18)

Supplementary Table 8. Anisotropic Displacement Parameters ($Å^2×10^3$) for perovskite (CDMA)$(MA)_2Pb_3I_{10}$. The Anisotropic displacement factor exponent takes the form: $-2\pi^2[h^2a^{*2}U_{11}+2hka^*b^*U_{12}+…]$.

Atom	U_{11}	U_{22}	U_{33}	U_{23}	U_{13}	U_{12}
$Pb^{(1)}$	11.6(8)	15.2(15)	29.4(14)	0.2(11)	-0.3(8)	0.3(7)
$Pb^{(2)}$	22.3(8)	23.3(9)	29.4(9)	-0.1(17)	-0.9(12)	-0.1(6)
$Pb^{(3)}$	25.3(12)	26(2)	36.0(16)	0.1(13)	-1.0(10)	-1.9(9)
$I^{(2)}$	26.1(17)	27(3)	49(3)	7(2)	2.4(17)	15.6(17)
$I^{(4)}$	38(2)	38(3)	20(2)	1(2)	-1.7(17)	15(2)
$I^{(3)}$	28.5(17)	28(3)	69(4)	11(3)	6(2)	16.1(17)
$I^{(1)}$	34(2)	56(4)	23(2)	2(2)	-3.6(17)	11(2)
$I^{(5)}$	29.1(12)	31.8(16)	48.5(15)	-4(3)	-6(2)	13.0(12)
$I^{(6)}$	28.4(13)	27.2(16)	48.2(15)	3(3)	-3(2)	-8.7(11)
$I^{(10)}$	36(2)	41(3)	41(3)	4(3)	-4.4(18)	-11(2)
$I^{(7)}$	41(2)	44(4)	32(3)	0(3)	-1.9(17)	-17(2)
$I^{(9)}$	35(2)	40(4)	46(3)	3(2)	-0.3(18)	-16(2)
$I^{(8)}$	37(2)	35(4)	83(4)	11(3)	-12(2)	-13(2)
$C^{(1)}$	70(30)	10(30)	60(30)	0(20)	20(30)	10(20)
$N^{(1)}$	70(30)	40(30)	60(30)	0(20)	10(20)	0(20)
$C^{(3)}$	40(16)	80(20)	64(15)	-19(15)	19(14)	-41(15)
$N^{(3)}$	40(20)	70(30)	50(20)	-20(20)	24(16)	-13(19)
$C^{(2)}$	30(30)	90(50)	70(40)	-20(30)	30(20)	0(30)
$C^{(7)}$	47(16)	90(20)	61(14)	-18(14)	13(12)	-44(14)
$C^{(5)}$	45(16)	90(20)	60(14)	-24(14)	17(13)	-41(14)
$C^{(8)}$	49(16)	100(20)	62(14)	-22(14)	8(13)	-43(14)
$C^{(6)}$	42(15)	100(20)	62(14)	-25(14)	11(13)	-41(14)
$C^{(4)}$	48(15)	88(19)	63(13)	-21(13)	15(12)	-44(13)
$C^{(9)}$	45(15)	101(19)	63(13)	-21(14)	7(12)	-42(14)
$C^{(10)}$	42(16)	100(20)	64(16)	-19(16)	4(14)	-42(16)
$N^{(4)}$	26(19)	100(30)	50(20)	0(20)	3(17)	-40(20)
$N^{(2)}$	30(20)	100(50)	80(40)	-20(30)	40(20)	0(20)

Supplementary Table 9. Bond Lengths for perovskite $(CDMA)(MA)_2Pb_3I_{10}$.

Atom	Atom	Length/Å	Atom	Atom	Length/Å
$Pb^{(1)}$	$I^{(2)1}$	3.171(5)	$I^{(2)}$	$Pb^{(1)3}$	3.171(4)
$Pb^{(1)}$	$I^{(2)}$	3.171(4)	$I^{(3)}$	$Pb^{(1)4}$	3.156(5)
$Pb^{(1)}$	$I^{(4)}$	3.315(5)	$I^{(5)}$	$Pb^{(2)4}$	3.188(3)
$Pb^{(1)}$	$I^{(3)}$	3.143(4)	$I^{(6)}$	$Pb^{(2)1}$	3.258(3)
$Pb^{(1)}$	$I^{(3)2}$	3.156(5)	$I^{(9)}$	$Pb^{(3)1}$	3.193(5)
$Pb^{(1)}$	$I^{(1)}$	3.097(5)	$I^{(8)}$	$Pb^{(3)2}$	3.126(5)
$Pb^{(2)}$	$I^{(4)}$	3.168(6)	$C^{(1)}$	$N^{(1)}$	1.44(9)
$Pb^{(2)}$	$I^{(5)2}$	3.188(3)	$C^{(3)}$	$N^{(3)}$	1.50(3)
$Pb^{(2)}$	$I^{(5)}$	3.162(3)	$C^{(3)}$	$C^{(4)}$	1.484(19)
$Pb^{(2)}$	$I^{(6)}$	3.220(3)	$C^{(2)}$	$N^{(2)}$	1.48(3)
$Pb^{(2)}$	$I^{(6)3}$	3.258(3)	$C^{(7)}$	$C^{(8)}$	1.505(19)
$Pb^{(2)}$	$I^{(7)}$	3.165(6)	$C^{(7)}$	$C^{(4)}$	1.524(19)
$Pb^{(3)}$	$I^{(10)}$	3.072(6)	$C^{(5)}$	$C^{(6)}$	1.51(3)
$Pb^{(3)}$	$I^{(7)}$	3.321(6)	$C^{(5)}$	$C^{(4)}$	1.52(2)
$Pb^{(3)}$	$I^{(9)3}$	3.193(5)	$C^{(8)}$	$C^{(9)}$	1.52(3)
$Pb^{(3)}$	$I^{(9)}$	3.181(5)	$C^{(6)}$	$C^{(9)}$	1.52(3)
$Pb^{(3)}$	$I^{(8)4}$	3.126(5)	$C^{(9)}$	$C^{(10)}$	1.494(19)
$Pb^{(3)}$	$I^{(8)}$	3.139(6)	$C^{(10)}$	$N^{(4)}$	1.48(3)

^{1}-1/2+X, 1/2-Y, +Z; ^{2}1/2+X, 3/2-Y, +Z; ^{3}1/2+X, 1/2-Y, +Z; ^{4}-1/2+X, 3/2-Y, +Z

Supplementary Table 10. Bond Angles for perovskite $(CDMA)(MA)_2Pb_3I_{10}$.

Atom	Atom	Atom	Angle/°	Atom	Atom	Atom	Angle/°
$I^{(2)1}$	$Pb^{(1)}$	$I^{(2)}$	89.47(7)	$I^{(10)}$	$Pb^{(3)}$	$I^{(8)}$	98.14(18)
$I^{(2)}$	$Pb^{(1)}$	$I^{(4)}$	84.81(14)	$I^{(10)}$	$Pb^{(3)}$	$I^{(8)4}$	92.53(17)
$I^{(2)1}$	$Pb^{(1)}$	$I^{(4)}$	85.37(15)	$I^{(9)}$	$Pb^{(3)}$	$I^{(7)}$	84.60(15)
$I^{(3)2}$	$Pb^{(1)}$	$I^{(2)}$	88.80(13)	$I^{(9)3}$	$Pb^{(3)}$	$I^{(7)}$	84.83(16)
$I^{(3)2}$	$Pb^{(1)}$	$I^{(2)1}$	174.65(19)	$I^{(9)}$	$Pb^{(3)}$	$I^{(9)3}$	88.97(8)
$I^{(3)}$	$Pb^{(1)}$	$I^{(2)}$	174.67(19)	$I^{(8)}$	$Pb^{(3)}$	$I^{(7)}$	89.64(16)
$I^{(3)}$	$Pb^{(1)}$	$I^{(2)1}$	91.37(13)	$I^{(8)4}$	$Pb^{(3)}$	$I^{(7)}$	89.45(17)
$I^{(3)2}$	$Pb^{(1)}$	$I^{(4)}$	89.42(16)	$I^{(8)}$	$Pb^{(3)}$	$I^{(9)3}$	87.63(14)
$I^{(3)}$	$Pb^{(1)}$	$I^{(4)}$	90.02(15)	$I^{(8)}$	$Pb^{(3)}$	$I^{(9)}$	173.6(2)
$I^{(3)}$	$Pb^{(1)}$	$I^{(3)2}$	89.89(7)	$I^{(8)4}$	$Pb^{(3)}$	$I^{(9)}$	92.57(14)
$I^{(1)}$	$Pb^{(1)}$	$I^{(2)}$	93.67(15)	$I^{(8)4}$	$Pb^{(3)}$	$I^{(9)3}$	173.9(2)
$I^{(1)}$	$Pb^{(1)}$	$I^{(2)1}$	87.16(16)	$I^{(8)4}$	$Pb^{(3)}$	$I^{(8)}$	90.26(8)
$I^{(1)}$	$Pb^{(1)}$	$I^{(4)}$	172.39(16)	$Pb^{(1)3}$	$I^{(2)}$	$Pb^{(1)}$	159.08(18)
$I^{(1)}$	$Pb^{(1)}$	$I^{(3)2}$	98.01(18)	$Pb^{(2)}$	$I^{(4)}$	$Pb^{(1)}$	176.16(17)
$I^{(1)}$	$Pb^{(1)}$	$I^{(3)}$	91.63(15)	$Pb^{(1)}$	$I^{(3)}$	$Pb^{(1)4}$	163.38(19)
$I^{(4)}$	$Pb^{(2)}$	$I^{(5)2}$	89.06(18)	$Pb^{(2)}$	$I^{(5)}$	$Pb^{(2)4}$	149.54(11)
$I^{(4)}$	$Pb^{(2)}$	$I^{(6)3}$	87.05(18)	$Pb^{(2)}$	$I^{(6)}$	$Pb^{(2)1}$	156.66(10)
$I^{(4)}$	$Pb^{(2)}$	$I^{(6)}$	90.21(18)	$Pb^{(2)}$	$I^{(7)}$	$Pb^{(3)}$	176.7(2)
$I^{(5)}$	$Pb^{(2)}$	$I^{(4)}$	92.45(18)	$Pb^{(3)}$	$I^{(9)}$	$Pb^{(3)1}$	157.9(2)
$I^{(5)}$	$Pb^{(2)}$	$I^{(5)2}$	91.72(5)	$Pb^{(3)2}$	$I^{(8)}$	$Pb^{(3)}$	165.1(2)
$I^{(5)2}$	$Pb^{(2)}$	$I^{(6)}$	174.57(9)	$C^{(4)}$	$C^{(3)}$	$N^{(3)}$	111(4)
$I^{(5)2}$	$Pb^{(2)}$	$I^{(6)3}$	86.58(8)	$C^{(8)}$	$C^{(7)}$	$C^{(4)}$	111(2)
$I^{(5)}$	$Pb^{(2)}$	$I^{(6)}$	93.68(8)	$C^{(6)}$	$C^{(5)}$	$C^{(4)}$	109(2)
$I^{(5)}$	$Pb^{(2)}$	$I^{(6)3}$	178.23(12)	$C^{(7)}$	$C^{(8)}$	$C^{(9)}$	109(2)
$I^{(5)}$	$Pb^{(2)}$	$I^{(7)}$	93.10(19)	$C^{(5)}$	$C^{(6)}$	$C^{(9)}$	112(2)
$I^{(6)}$	$Pb^{(2)}$	$I^{(6)3}$	88.02(4)	$C^{(3)}$	$C^{(4)}$	$C^{(7)}$	112(2)
$I^{(7)}$	$Pb^{(2)}$	$I^{(4)}$	174.17(9)	$C^{(3)}$	$C^{(4)}$	$C^{(5)}$	112(2)
$I^{(7)}$	$Pb^{(2)}$	$I^{(5)2}$	89.00(18)	$C^{(5)}$	$C^{(4)}$	$C^{(7)}$	111(2)
$I^{(7)}$	$Pb^{(2)}$	$I^{(6)3}$	87.34(19)	$C^{(6)}$	$C^{(9)}$	$C^{(8)}$	112(2)
$I^{(7)}$	$Pb^{(2)}$	$I^{(6)}$	91.21(18)	$C^{(10)}$	$C^{(9)}$	$C^{(8)}$	110(2)
$I^{(10)}$	$Pb^{(3)}$	$I^{(7)}$	171.96(16)	$C^{(10)}$	$C^{(9)}$	$C^{(6)}$	112(2)
$I^{(10)}$	$Pb^{(3)}$	$I^{(9)3}$	93.42(16)	$N^{(4)}$	$C^{(10)}$	$C^{(9)}$	114(3)
$I^{(10)}$	$Pb^{(3)}$	$I^{(9)}$	87.53(16)				

^{1}-1/2+X, 1/2-Y, +Z; ^{2}1/2+X, 3/2-Y, +Z; ^{3}1/2+X, 1/2-Y, +Z; ^{4}-1/2+X, 3/2-Y, +Z

Supplementary Table 11. Crystal data and structure refinement for perovskite $(CDMA)(MA)Pb_2I_7$.

**Parameters**	**Perovskite**
CCDC number	2357880
Empirical formula	$(CDMA)(MA)Pb_2I_7$
Formula weight	1479.01
Temperature/*K*	273.15
Crystal system	orthorhombic
Space group	$Pca2_1$
a/Å	8.8151(4)
b/Å	8.7927(5)
c/Å	36.583(2)
α/°	90
β/°	90
γ/°	90
Volume/Å3	2835.5(3)
Z	4
ρ_{calc} g/cm^3	3.465
μ mm^{-1}	19.48
F(000)	2544
Radiation	Mo Kα (λ = 0.71073)
2Θ range for data collection/°	4.632 to 49.992
Index ranges	$-9 \leq h \leq 9$, $-7 \leq k \leq 10$, $-43 \leq l \leq 43$
Reflections collected	19458
Independent reflections	4760 [R_{int} = 0.0412, R_{sigma} = 0.0424]
Data/restraints/parameters	4760/198/194
Goodness-of-fit on F2	1.072
Final R indexes [I>=2σ (I)]	R_1 = 0.0596, wR_2 = 0.1728
Final R indexes [all data]	R_1 = 0.0639, wR_2 = 0.1765
Largest diff. peak/hole / e Å^{-3}	3.07/-3.06
Flack parameter	0.511(14)

Supplementary Table 12. Fractional Atomic Coordinates (×10^4) and Equivalent Isotropic Displacement Parameters (Å^2×10^3) for perovskite (CDMA)(MA)Pb_2I_7. U_{eq} is defined as 1/3 of of the trace of the orthogonalised U_{IJ} tensor.

**Atom**	***x***	***y***	***z***	***U*(eq)**
Pb(1)	1673.8(17)	2502.5(14)	4964.9(4)	16.7(4)
Pb(2)	1718.8(18)	2492.1(13)	6751.9(4)	17.0(4)
I(6)	1936(3)	2189(3)	7607.1(8)	25.5(6)
I(5)	-302(3)	5494(3)	6727.9(12)	38.6(9)
I(4)	-1323(3)	540(3)	6706.2(10)	25.8(7)
I(3)	1642(2)	2462(4)	5860.4(13)	27.1(5)
I(1)	1868(3)	2841(3)	4113.3(9)	24.3(6)
I(2)	4639(3)	464(3)	4986.4(11)	29.2(7)
N(3)	9120(40)	1730(30)	2389(8)	20(5)
C(3)	7340(40)	2480(40)	3750(9)	30(4)
I(7)	3634(3)	5556(3)	5004.6(10)	26.1(7)
C(7)	8830(40)	1730(40)	3205(10)	29(4)
C(4)	8240(50)	1250(40)	3575(10)	31(4)
C(2)	6900(40)	1910(40)	4126(9)	32(4)
C(5)	6080(40)	3050(40)	3509(10)	32(4)
C(9)	8380(40)	2940(40)	2613(9)	27(4)
C(6)	6740(50)	3580(40)	3151(10)	31(4)
N(2)	5920(40)	3020(40)	4325(10)	30(6)
C(8)	7600(40)	2350(40)	2957(8)	28(4)
N	-2460(50)	3310(40)	5969(9)	59(11)
C(1)	-3770(60)	2490(70)	5808(15)	72(13)

Supplementary Table 13. Anisotropic Displacement Parameters (Å^2×10^3) for perovskite (CDMA)(MA)Pb_2I_7. The Anisotropic displacement factor exponent takes the form: $-2\pi^2[h^2a^{*2}U_{11}+2hka^*b^*U_{12}+\ldots]$.

Atom	U_{11}	U_{22}	U_{33}	U_{23}	U_{13}	U_{12}
Pb(1)	13.1(8)	15.8(9)	21.3(10)	0.6(6)	-3.5(6)	0.0(5)
Pb(2)	18.4(9)	12.1(8)	20.6(10)	0.7(6)	-4.7(6)	0.2(5)
I(6)	31.4(15)	30.9(13)	14.1(14)	-2.6(13)	-1.3(12)	2.6(11)
I(5)	29.0(16)	39.4(16)	48(2)	-11.5(17)	-13.2(15)	14.7(12)
I(4)	23.2(15)	20.3(12)	34(2)	3.9(12)	-0.8(12)	-12.5(10)
I(3)	32.3(12)	31.4(11)	17.5(9)	0.3(6)	-2.2(15)	-9.1(16)
I(1)	26.1(14)	24.1(12)	22.7(16)	-1.5(14)	-4.6(12)	0.9(10)
I(2)	26.8(15)	14.0(11)	47(2)	10.4(13)	7.5(14)	16.2(11)
N(3)	36(12)	1(9)	21(11)	3(9)	5(10)	2(9)
C(3)	32(8)	28(7)	29(7)	-4(6)	1(6)	0(6)
I(7)	22.2(15)	30.3(14)	25.9(18)	-2.4(12)	0.5(12)	-9.3(11)
C(7)	33(8)	26(7)	29(7)	-4(6)	1(6)	3(6)
C(4)	33(8)	30(7)	30(7)	-4(6)	0(6)	2(6)
C(2)	34(9)	32(9)	29(8)	-3(8)	1(8)	2(8)
C(5)	33(8)	30(7)	31(7)	-2(7)	2(7)	0(7)
C(9)	32(9)	24(8)	25(8)	-5(7)	-1(8)	4(8)
C(6)	34(8)	29(7)	29(7)	-5(6)	-1(6)	2(6)
N(2)	31(13)	31(12)	27(12)	-5(11)	5(11)	-15(10)
C(8)	32(8)	26(7)	27(7)	-5(6)	1(6)	1(6)
N	110(30)	39(19)	29(18)	9(15)	45(19)	27(19)
C(1)	120(30)	60(20)	40(30)	16(19)	30(20)	20(20)

Supplementary Table 14. Bond Lengths for perovskite (CDMA)(MA)Pb_2I_7.

Atom	Atom	Length/Å	Atom	Atom	Length/Å
Pb(1)	I(3)	3.276(5)	I(2)	Pb(1)4	3.167(3)
Pb(1)	I(1)	3.134(4)	N(3)	C(9)	1.49(2)
Pb(1)	I(2)	3.170(3)	C(3)	C(4)	1.49(2)
Pb(1)	I(2)1	3.167(3)	C(3)	C(2)	1.51(3)
Pb(1)	I(7)	3.196(3)	C(3)	C(5)	1.50(3)
Pb(1)	I(7)2	3.181(3)	I(7)	Pb(1)3	3.181(3)
Pb(2)	I(6)	3.146(3)	C(7)	C(4)	1.51(3)
Pb(2)	I(5)	3.186(3)	C(7)	C(8)	1.51(3)
Pb(2)	I(5)3	3.168(3)	C(2)	N(2)	1.49(2)
Pb(2)	I(4)	3.188(3)	C(5)	C(6)	1.51(3)
Pb(2)	I(4)4	3.180(3)	C(9)	C(8)	1.52(2)
Pb(2)	I(3)	3.262(5)	C(6)	C(8)	1.50(2)
I(5)	Pb(2)2	3.168(3)	N	C(1)	1.48(3)
I(4)	Pb(2)1	3.180(3)			

1-1/2+X, -Y, +Z; 2-1/2+X, 1-Y, +Z; 31/2+X, 1-Y, +Z; 41/2+X, -Y, +Z

Supplementary Table 15. Bond Angles for perovskite (CDMA)(MA)Pb_2I_7.

Atom	Atom	Atom	Angle/°	Atom	Atom	Atom	Angle/°
$I^{(1)}$	$Pb^{(1)}$	$I^{(3)}$	174.49(9)	$I^{(5)}$	$Pb^{(2)}$	$I^{(3)}$	88.15(11)
$I^{(1)}$	$Pb^{(1)}$	$I^{(2)1}$	97.71(11)	$I^{(5)3}$	$Pb^{(2)}$	$I^{(3)}$	89.66(10)
$I^{(1)}$	$Pb^{(1)}$	$I^{(2)}$	91.91(10)	$I^{(4)4}$	$Pb^{(2)}$	$I^{(5)}$	175.28(14)
$I^{(1)}$	$Pb^{(1)}$	$I^{(7)}$	86.32(10)	$I^{(4)4}$	$Pb^{(2)}$	$I^{(4)}$	90.14(4)
$I^{(1)}$	$Pb^{(1)}$	$I^{(7)2}$	92.32(10)	$I^{(4)4}$	$Pb^{(2)}$	$I^{(3)}$	87.24(10)
$I^{(2)1}$	$Pb^{(1)}$	$I^{(3)}$	87.78(10)	$I^{(4)}$	$Pb^{(2)}$	$I^{(3)}$	85.74(9)
$I^{(2)}$	$Pb^{(1)}$	$I^{(3)}$	88.62(10)	$Pb^{(2)2}$	$I^{(5)}$	$Pb^{(2)}$	157.77(13)
$I^{(2)1}$	$Pb^{(1)}$	$I^{(2)}$	90.04(4)	$Pb^{(2)1}$	$I^{(4)}$	$Pb^{(2)}$	154.84(11)
$I^{(2)}$	$Pb^{(1)}$	$I^{(7)}$	91.61(8)	$Pb^{(2)}$	$I^{(3)}$	$Pb^{(1)}$	178.00(9)
$I^{(2)}$	$Pb^{(1)}$	$I^{(7)2}$	175.52(13)	$Pb^{(1)4}$	$I^{(2)}$	$Pb^{(1)}$	158.76(11)
$I^{(2)1}$	$Pb^{(1)}$	$I^{(7)2}$	87.92(8)	$C^{(4)}$	$C^{(3)}$	$C^{(2)}$	106(2)
$I^{(2)1}$	$Pb^{(1)}$	$I^{(7)}$	175.60(13)	$C^{(4)}$	$C^{(3)}$	$C^{(5)}$	113(3)
$I^{(7)}$	$Pb^{(1)}$	$I^{(3)}$	88.19(9)	$C^{(5)}$	$C^{(3)}$	$C^{(2)}$	117(3)
$I^{(7)2}$	$Pb^{(1)}$	$I^{(3)}$	87.31(10)	$Pb^{(1)3}$	$I^{(7)}$	$Pb^{(1)}$	154.73(11)
$I^{(7)2}$	$Pb^{(1)}$	$I^{(7)}$	90.14(4)	$C^{(4)}$	$C^{(7)}$	$C^{(8)}$	113(3)
$I^{(6)}$	$Pb^{(2)}$	$I^{(5)}$	97.57(12)	$C^{(3)}$	$C^{(4)}$	$C^{(7)}$	111(3)
$I^{(6)}$	$Pb^{(2)}$	$I^{(5)3}$	91.41(11)	$C^{(3)}$	$C^{(2)}$	$N^{(2)}$	112(3)
$I^{(6)}$	$Pb^{(2)}$	$I^{(4)4}$	87.03(10)	$C^{(3)}$	$C^{(5)}$	$C^{(6)}$	109(3)
$I^{(6)}$	$Pb^{(2)}$	$I^{(4)}$	93.30(10)	$N^{(3)}$	$C^{(9)}$	$C^{(8)}$	114(2)
$I^{(6)}$	$Pb^{(2)}$	$I^{(3)}$	174.18(9)	$C^{(8)}$	$C^{(6)}$	$C^{(5)}$	112(3)
$I^{(5)3}$	$Pb^{(2)}$	$I^{(5)}$	89.98(4)	$C^{(7)}$	$C^{(8)}$	$C^{(9)}$	107(3)
$I^{(5)3}$	$Pb^{(2)}$	$I^{(4)}$	175.21(14)	$C^{(6)}$	$C^{(8)}$	$C^{(7)}$	110(3)
$I^{(5)}$	$Pb^{(2)}$	$I^{(4)}$	88.54(8)	$C^{(6)}$	$C^{(8)}$	$C^{(9)}$	112(2)
$I^{(5)3}$	$Pb^{(2)}$	$I^{(4)4}$	90.97(8)				

^{1}-1/2+X, -Y, +Z; ^{2}-1/2+X, 1-Y, +Z; ^{3}1/2+X, 1-Y, +Z; ^{4}1/2+X, -Y, +Z

Supplementary Table 16. Crystal data and structure refinement for perovskite (CDMA)PbI_4.

**Parameters**	**Perovskite**
CCDC number	2357879
Empirical formula	(CDMA)PbI_4
Formula weight	859.05
Temperature/K	273.15
Crystal system	monoclinic
Space group	$P2_1/c$
a/Å	12.1827(10)
b/Å	8.7410(6)
c/Å	8.7452(7)
α/°	90
β/°	95.945(3)
γ/°	90
Volume/Å3	926.26(12)
Z	2
ρ_{calc} g/cm^3	3.08
μ mm^{-1}	15.754
F(000)	752
Radiation	Mo Kα (λ = 0.71073)
2Θ range for data collection/°	5.746 to 56.566
Index ranges	$-16 \leq h \leq 16$, $-9 \leq k \leq 11$, $-8 \leq l \leq 11$
Reflections collected	8147
Independent reflections	2281 [R_{int} = 0.0586, R_{sigma} = 0.0531]
Data/restraints/parameters	2281/0/71
Goodness-of-fit on F2	1.048
Final R indexes [I>=2σ (I)]	R_1 = 0.0303, wR_2 = 0.0801
Final R indexes [all data]	R_1 = 0.0350, wR_2 = 0.0828
Largest diff. peak/hole / e Å^{-3}	1.63/-1.14

Supplementary Table 17. Fractional Atomic Coordinates (×10^4) and Equivalent Isotropic Displacement Parameters (Å^2×10^3) for perovskite (CDMA)PbI_4. U_{eq} is defined as 1/3 of of the trace of the orthogonalised U_{IJ} tensor.

Atom	*x*	*y*	*z*	U(eq)
Pb(1)	5000	5000	5000	18.57(10)
I(1)	4980.1(3)	6910.0(4)	8082.5(5)	27.38(13)
I(2)	7639.6(3)	5264.4(5)	5231.3(5)	27.48(12)
N(1)	7118(4)	5788(6)	1129(6)	30.0(12)
C(1)	7713(7)	4589(10)	329(11)	46.2(19)
C(3)	9567(6)	5678(10)	1324(9)	48(2)
C(4)	10632(6)	6173(9)	891(9)	44.0(19)
C(2)	8790(6)	5110(7)	-60(8)	31.9(17)

Supplementary Table 18. Anisotropic Displacement Parameters (Å^2×10^3) for perovskite $(CDMA)_2PbI_4$. The Anisotropic displacement factor exponent takes the form: $-2\pi^2[h^2a^{*2}U_{11}+2hka^*b^*U_{12}+\ldots]$.

Atom	U_{11}	U_{22}	U_{33}	U_{23}	U_{13}	U_{12}
Pb(1)	23.85(18)	16.44(17)	15.50(17)	-0.16(10)	2.50(12)	-0.9(1)
I(1)	38.8(2)	22.1(2)	21.8(2)	-8.60(14)	5.98(17)	-6.41(15)
I(2)	24.4(2)	28.7(2)	28.9(2)	0.12(16)	0.69(17)	3.00(15)
N(1)	25(3)	34(3)	31(3)	3(2)	6(2)	3(2)
C(1)	48(5)	42(4)	51(5)	-12(4)	15(4)	4(4)
C(3)	32(4)	60(5)	48(5)	-21(4)	-12(3)	13(4)
C(4)	34(4)	51(5)	46(5)	-23(4)	2(3)	7(3)
C(2)	31(4)	28(4)	36(5)	-4(3)	4(3)	3(2)

Supplementary Table 19. Bond Lengths for (CDMA)PbI_4.

Atom	Atom	Length/Å	Atom	Atom	Length/Å
$Pb^{(1)}$	$I^{(1)}$	3.1731(4)	$N^{(1)}$	$C^{(1)}$	1.490(9)
$Pb^{(1)}$	$I^{(1)1}$	3.1731(4)	$C^{(1)}$	$C^{(2)}$	1.462(10)
$Pb^{(1)}$	$I^{(1)2}$	3.1779(4)	$C^{(3)}$	$C^{(4)}$	1.455(11)
$Pb^{(1)}$	$I^{(1)3}$	3.1779(4)	$C^{(3)}$	$C^{(2)}$	1.539(10)
$Pb^{(1)}$	$I^{(2)}$	3.2095(5)	$C^{(4)}$	$C^{(2)5}$	1.545(9)
$Pb^{(1)}$	$I^{(2)1}$	3.2095(5)	$C^{(2)}$	$C^{(4)5}$	1.545(9)
$I^{(1)}$	$Pb^{(1)4}$	3.1779(4)			

[1]1-X, 1-Y, 1-Z; [2]+X, 3/2-Y, -1/2+Z; [3]1-X, -1/2+Y, 3/2-Z; [4]1-X, 1/2+Y, 3/2-Z; [5]2-X, 1-Y, -Z

Supplementary Table 20. Bond Angles for (CDMA)PbI_4.

Atom	Atom	Atom	Angle/°	Atom	Atom	Atom	Angle/°
$I^{(1)}$	$Pb^{(1)}$	$I^{(1)1}$	180	$I^{(1)1}$	$Pb^{(1)}$	$I^{(2)}$	89.751(11)
$I^{(1)}$	$Pb^{(1)}$	$I^{(1)2}$	89.955(6)	$I^{(1)3}$	$Pb^{(1)}$	$I^{(2)1}$	94.307(11)
$I^{(1)1}$	$Pb^{(1)}$	$I^{(1)2}$	90.045(6)	$I^{(1)}$	$Pb^{(1)}$	$I^{(2)}$	90.249(11)
$I^{(1)1}$	$Pb^{(1)}$	$I^{(1)3}$	89.955(6)	$I^{(2)}$	$Pb^{(1)}$	$I^{(2)1}$	180
$I^{(1)}$	$Pb^{(1)}$	$I^{(1)3}$	90.045(6)	$Pb^{(1)}$	$I^{(1)}$	$Pb^{(1)4}$	153.528(14)
$I^{(1)3}$	$Pb^{(1)}$	$I^{(1)2}$	180	$C^{(2)}$	$C^{(1)}$	$N^{(1)}$	112.5(6)
$I^{(1)1}$	$Pb^{(1)}$	$I^{(2)1}$	90.248(11)	$C^{(4)}$	$C^{(3)}$	$C^{(2)}$	112.6(7)
$I^{(1)2}$	$Pb^{(1)}$	$I^{(2)}$	94.307(11)	$C^{(3)}$	$C^{(4)}$	$C^{(2)5}$	111.6(6)
$I^{(1)}$	$Pb^{(1)}$	$I^{(2)1}$	89.752(11)	$C^{(1)}$	$C^{(2)}$	$C^{(3)}$	114.3(7)
$I^{(1)3}$	$Pb^{(1)}$	$I^{(2)}$	85.693(11)	$C^{(1)}$	$C^{(2)}$	$C^{(4)5}$	110.3(6)
$I^{(1)2}$	$Pb^{(1)}$	$I^{(2)1}$	85.693(11)	$C^{(3)}$	$C^{(2)}$	$C^{(4)5}$	109.3(6)

[1]1-X, 1-Y, 1-Z; [2]1-X, -1/2+Y, 3/2-Z; [3]+X, 3/2-Y, -1/2+Z; [4]1-X, 1/2+Y, 3/2-Z; [5]2-X, 1-Y, -Z

Supplementary References

1. Kresse, G. & Furthmuller, J. Efficient iterative schemes for ab initio total-energy calculations using a plane-wave basis set. *Phys Rev B Condens Matter* **54**, 11169-11186 (1996).
2. Blochl, P. E. Projector augmented-wave method. *Phys Rev B Condens Matter* **50**, 17953-17979 (1994).
3. Monkhorst, H. J. & Pack, J. D. Special points for Brillouin-zone integrations. *Physical Review B* **13**, 5188-5192 (1976).
4. Lv, G. *et al.* Multiple-Noncovalent-Interaction-Stabilized Layered Dion-Jacobson Perovskite for Efficient Solar Cells. *Nano Lett.* **21**, 5788-5797 (2021).
5. Fu, P. *et al.* Dion-Jacobson and Ruddlesden-Popper double-phase 2D perovskites for solar cells. *Nano Energy* **88**, 106249 (2021).
6. Zhang, X. *et al.* Film Formation Control for High Performance Dion–Jacobson 2D Perovskite Solar Cells. *Adv. Energy Mater.* **11**, 2002733 (2021).
7. Xu, Z. *et al.* Highly Efficient and Stable Dion-Jacobson Perovskite Solar Cells Enabled by Extended pi-Conjugation of Organic Spacer. *Adv. Mater.* **33**, e2105083 (2021).
8. Wu, H. *et al.* Merged interface construction toward ultra-low Voc loss in inverted two-dimensional Dion–Jacobson perovskite solar cells with efficiency over 18%. *J. Mater. Chem. A* **9**, 12566-12573 (2021).
9. Ahmad, S. *et al.* Formamidinium-incorporated Dion-Jacobson phase 2D perovskites for highly efficient and stable photovoltaics. *Journal of Energy Chemistry* **57**, 632-638 (2021).
10. Wang, H. *et al.* Efficient Slantwise Aligned Dion-Jacobson Phase Perovskite Solar Cells Based on Trans-1,4-Cyclohexanediamine. *Small* **16**, e2003098 (2020).
11. Ke, W. *et al.* Compositional and Solvent Engineering in Dion-Jacobson 2D Perovskites Boosts Solar Cell Efficiency and Stability. *Adv. Energy Mater.* **9**, 1803384 (2019).
12. Cheng, L. *et al.* Tailoring Interlayer Spacers for Efficient and Stable Formamidinium-Based Low-Dimensional Perovskite Solar Cells. *Adv. Mater.*, e2106380 (2021).
13. Yukta *et al.* Thiocyanate-Passivated Diaminonaphthalene-Incorporated Dion-Jacobson Perovskite for Highly Efficient and Stable Solar Cells. *ACS Appl. Mater. Interfaces* **14**, 850-860 (2022).
14. Cheng, L. *et al.* Highly Thermostable and Efficient Formamidinium-Based Low-Dimensional Perovskite Solar Cells. *Angew. Chem. Int. Ed.* **60**, 856-864 (2021).
15. Lu, D. *et al.* Thiophene-Based Two-Dimensional Dion-Jacobson Perovskite Solar Cells with over 15% Efficiency. *J. Am. Chem. Soc.* **142**, 11114-11122 (2020).
16. Li, F. *et al.* Vertical Orientated Dion–Jacobson Quasi-2D Perovskite Film with Improved Photovoltaic Performance and Stability. *Small Methods* **4**, 1900831 (2019).
17. Su, P. *et al.* Crystal Orientation Modulation and Defect Passivation for Efficient and Stable Methylammonium-Free Dion-Jacobson Quasi-2D Perovskite Solar Cells. *ACS Appl. Mater. Interfaces* **13**, 29567-29575 (2021).
18. Zheng, Y. *et al.* Oriented and Uniform Distribution of Dion–Jacobson Phase Perovskites Controlled by Quantum Well Barrier Thickness. *Solar RRL* **3**, 1900090 (2019).
19. Yu, S. *et al.* Nonconfinement Structure Revealed in Dion-Jacobson Type Quasi-2D Perovskite Expedites Interlayer Charge Transport. *Small* **15**, e1905081 (2019).
20. Liu, Y. C. *et al.* Recognizing the Importance of Fast Nonisothermal Crystallization for High-

Performance Two-Dimensional Dion-Jacobson Perovskite Solar Cells with High Fill Factors: A Comprehensive Mechanistic Study. *J. Am. Chem. Soc.* **144**, 14897-14906 (2022).

21. Guo, X. *et al.* Suppressed Phase Segregation in High-Humidity-Processed Dion–Jacobson Perovskite Solar Cells Toward High Efficiency and Stability. *Solar RRL* **5**, 2100555 (2021).
22. Gao, L. *et al.* m-Phenylenediammonium as a New Spacer for Dion-Jacobson Two-Dimensional Perovskites. *J. Am. Chem. Soc.* **143**, 12063-12073 (2021).
23. Wang, D., Chen, S.-C. & Zheng, Q. Enhancing the efficiency and stability of two-dimensional Dion–Jacobson perovskite solar cells using a fluorinated diammonium spacer. *J. Mater. Chem. A* **9**, 11778-11786 (2021).
24. Wang, J. *et al.* Templated growth of oriented layered hybrid perovskites on 3D-like perovskites. *Nat. Commun.* **11**, 582 (2020).
25. Wu, G., Ahmad, N. & Zhang, Y. High-efficiency of 15.47% for two-dimensional perovskite solar cells processed by blade coating with non-thermal assistance. *J. Mater. Chem. C* **9**, 9851-9858 (2021).
26. Meng, K. *et al.* Humidity-Insensitive, Large-Area-Applicable, Hot-Air-Assisted Ambient Fabrication of 2D Perovskite Solar Cells. *Adv. Mater.* **35**, e2209712 (2023).
27. Jang, G. *et al.* Elucidation of the Formation Mechanism of Highly Oriented Multiphase Ruddlesden–Popper Perovskite Solar Cells. *ACS Energy Lett.* **6**, 249-260 (2020).
28. Zhang, Y. & Park, N.-G. Quasi-Two-Dimensional Perovskite Solar Cells with Efficiency Exceeding 22%. *ACS Energy Lett.* **7**, 757-765 (2022).
29. Xu, Z. *et al.* Phase Distribution and Carrier Dynamics in Multiple-Ring Aromatic Spacer-Based Two-Dimensional Ruddlesden-Popper Perovskite Solar Cells. *ACS Nano* **14**, 4871-4881 (2020).
30. Lai, H. *et al.* Organic-Salt-Assisted Crystal Growth and Orientation of Quasi-2D Ruddlesden-Popper Perovskites for Solar Cells with Efficiency over 19. *Adv. Mater.* **32**, e2001470 (2020).
31. Ma, C., Shen, D., Ng, T. W., Lo, M. F. & Lee, C. S. 2D Perovskites with Short Interlayer Distance for High-Performance Solar Cell Application. *Adv. Mater.* **30**, e1800710 (2018).
32. Shao, M. *et al.* Over 21% Efficiency Stable 2D Perovskite Solar Cells. *Adv. Mater.* **34**, e2107211 (2022).
33. Shi, J. *et al.* Fluorinated Low-Dimensional Ruddlesden-Popper Perovskite Solar Cells with over 17% Power Conversion Efficiency and Improved Stability. *Adv. Mater.* **31**, e1901673 (2019).
34. Yang, Y. *et al.* Defect Suppression in Oriented 2D Perovskite Solar Cells with Efficiency over 18% via Rerouting Crystallization Pathway. *Adv. Energy Mater.* **11**, 2002966 (2021).
35. Fu, W. *et al.* Tailoring the Functionality of Organic Spacer Cations for Efficient and Stable Quasi-2D Perovskite Solar Cells. *Adv. Funct. Mater.* **29**, 1900221 (2019).
36. Lian, X. *et al.* Stable Quasi-2D Perovskite Solar Cells with Efficiency over 18% Enabled by Heat–Light Co-Treatment. *Adv. Funct. Mater.* **30**, 2004188 (2020).
37. Wang, Z. *et al.* Spacer Cation Tuning Enables Vertically Oriented and Graded Quasi-2D Perovskites for Efficient Solar Cells. *Adv. Funct. Mater.* **31**, 2008404 (2020).
38. Lian, X. *et al.* The Second Spacer Cation Assisted Growth of a 2D Perovskite Film with Oriented Large Grain for Highly Efficient and Stable Solar Cells. *Angew. Chem. Int. Ed.* **58**, 9409-9413 (2019).
39. Liang, J. *et al.* A finely regulated quantum well structure in quasi-2D Ruddlesden–Popper perovskite solar cells with efficiency exceeding 20%. *Energy Environ. Sci.* **15**, 296-310 (2022).
40. Lu, J. *et al.* Formamidinium-based Ruddlesden–Popper perovskite films fabricated via two-step

sequential deposition: quantum well formation, physical properties and film-based solar cells. *Energy Environ. Sci.* **15**, 1144-1155 (2022).
41. Yang, Y. *et al.* Universal approach toward high-efficiency two-dimensional perovskite solar cells via a vertical-rotation process. *Energy Environ. Sci.* **13**, 3093-3101 (2020).
42. Zhang, X. *et al.* Stable high efficiency two-dimensional perovskite solar cells via cesium doping. *Energy Environ. Sci.* **10**, 2095-2102 (2017).
43. Xi, J. *et al.* Alternative Organic Spacers for More Efficient Perovskite Solar Cells Containing Ruddlesden-Popper Phases. *J. Am. Chem. Soc.* **142**, 19705-19714 (2020).
44. Zhou, N. *et al.* Exploration of Crystallization Kinetics in Quasi Two-Dimensional Perovskite and High Performance Solar Cells. *J. Am. Chem. Soc.* **140**, 459-465 (2018).
45. Zhang, J. *et al.* Uniform Permutation of Quasi-2D Perovskites by Vacuum Poling for Efficient, High-Fill-Factor Solar Cells. *Joule* **3**, 3061-3071 (2019).
46. Tsai, H. *et al.* High-efficiency two-dimensional Ruddlesden-Popper perovskite solar cells. *Nature* **536**, 312-316 (2016).
47. Liang, C. *et al.* Two-dimensional Ruddlesden–Popper layered perovskite solar cells based on phase-pure thin films. *Nature Energy* **6**, 38-45 (2021).
48. Ren, H. *et al.* Efficient and stable Ruddlesden–Popper perovskite solar cell with tailored interlayer molecular interaction. *Nat. Photon.* **14**, 154-163 (2020).
49. Han, C. *et al.* Tailoring Phase Alignment and Interfaces via Polyelectrolyte Anchoring Enables Large-Area 2D Perovskite Solar Cells. *Angew. Chem. Int. Ed.*, e202205111 (2022).
50. Li, P. *et al.* Low-Dimensional Perovskites with Diammonium and Monoammonium Alternant Cations for High-Performance Photovoltaics. *Adv. Mater.* **31**, e1901966 (2019).
51. Zhao, X., Liu, T., Kaplan, A. B., Yao, C. & Loo, Y. L. Accessing Highly Oriented Two-Dimensional Perovskite Films via Solvent-Vapor Annealing for Efficient and Stable Solar Cells. *Nano Lett.* **20**, 8880-8889 (2020).
52. Wu, H. *et al.* Additive-Assisted Hot-Casting Free Fabrication of Dion–Jacobson 2D Perovskite Solar Cell with Efficiency Beyond 16%. *Solar RRL* **4**, 2000087 (2020).
53. Li, Z. *et al.* Organometallic-functionalized interfaces for highly efficient inverted perovskite solar cells. *Science* **376**, 416-420 (2022).
54. Kim, G. *et al.* Impact of strain relaxation on performance of alpha-formamidinium lead iodide perovskite solar cells. *Science* **370**, 108-112 (2020).
55. Hui, W. *et al.* Stabilizing black-phase formamidinium perovskite formation at room temperature and high humidity. *Science* **371**, 1359-1364 (2021).
56. Min, H. *et al.* Perovskite solar cells with atomically coherent interlayers on SnO_2 electrodes. *Nature* **598**, 444-450 (2021).
57. Jeong, J. *et al.* Pseudo-halide anion engineering for alpha-$FAPbI_3$ perovskite solar cells. *Nature* **592**, 381-385 (2021).
58. Bai, S. *et al.* Planar perovskite solar cells with long-term stability using ionic liquid additives. *Nature* **571**, 245-250 (2019).
59. Jung, E. H. *et al.* Efficient, stable and scalable perovskite solar cells using poly(3-hexylthiophene). *Nature* **567**, 511-515 (2019).
60. Li, Z. *et al.* 24.64%-Efficiency MA-Free Perovskite Solar Cell with Voc of 1.19 V Enabled by a Hinge-Type Fluorine-Rich Complex. *Adv. Funct. Mater.* **33**, 2212606 (2023).
61. Li, Y. *et al.* High-efficiency robust perovskite solar cells on ultrathin flexible substrates. *Nat.*

Commun. **7**, 10214 (2016).

62. Song, J. *et al.* Unraveling the Crystallization Kinetics of 2D Perovskites with Sandwich-Type Structure for High-Performance Photovoltaics. *Adv. Mater.* **32**, e2002784 (2020).
