# Supplementary material for: Ultrastable and efficient slight-interlayer-displacement 2D Dion-Jacobson perovskite solar cells
Source: Nat Commun. 2024 Jul 8;15:5709. doi: 10.1038/s41467-024-50018-4 (PMC11231157; doi:10.1038/s41467-024-50018-4)
Supplement: Supplementary file 3 — Description of Additional Supplementary Files [file 41467_2024_50018_MOESM3_ESM.pdf]

Description of Additional Supplementary Files

File name: Supplementary Data 1
Description: CIF file of the single crystals (1,4-cyclohexanedimethanammonium)(methylammonium)$_{n-1}$Pb$_n$I$_{3n+1}$ (n = 1-3).

File name: Supplementary Data 2
Description: CheckCIF file of the single crystals (1,4-cyclohexanedimethanammonium)(methylammonium)$_{n-1}$Pb$_n$I$_{3n+1}$ (n = 1-3).

File name: Supplementary Data 3
Description: Optimized computational models of the DFT calculation used for evaluating the formation energies of the reaction between the DJ perovskites (n = 1 PDMA- and CDMA-based perovskites) and water molecules.

File name: Supplementary Movie 1
Description: The dynamic dissolving process of a water droplet on the film surface of the CDMA-based perovskite film.

File name: Supplementary Movie 2
Description: The dynamic dissolving process of a water droplet on the film surface of the PDMA-based perovskite film.
