# Supplementary material for: Ultrastable and efficient slight-interlayer-displacement 2D Dion-Jacobson perovskite solar cells
Source: Nat Commun. 2024 Jul 8;15:5709. doi: 10.1038/s41467-024-50018-4 (PMC11231157; doi:10.1038/s41467-024-50018-4)

checkCIF/PLATON report

You have not supplied any structure factors. As a result the full set of tests cannot be run.

THIS REPORT IS FOR GUIDANCE ONLY. IF USED AS PART OF A REVIEW PROCEDURE FOR PUBLICATION, IT SHOULD NOT REPLACE THE EXPERTISE OF AN EXPERIENCED CRYSTALLOGRAPHIC REFEREE.

No syntax errors found. CIF dictionary Interpreting this report

Datablock: WeichuanZhang_CDMA-N1

Bond precision:	C-C = 0.0105 A		Wavelength=0.71073
Cell:	a=12.1827(10)	b=8.7410(6)	c=8.7452(7)
	alpha=90	beta=95.945(3)	gamma=90
Temperature:	273 K		

	Calculated	Reported
Volume	926.26(12)	926.26(12)
Space group	P 21/c	P 1 21/c 1
Hall group	-P 2ybc	-P 2ybc
Moiety formula	I4 Pb, 2(C4 H10 N)	I4 Pb, C8 H20 N2
Sum formula	C8 H20 I4 N2 Pb	C8 H20 I4 N2 Pb
Mr	859.06	859.05
Dx,g cm-3	3.080	3.080
Z	2	2
Mu (mm-1)	15.754	15.754
F000	752.0	752.0
F000’	742.18	
h,k,lmax	16,11,11	16,11,11
Nref	2292	2281
Tmin,Tmax		0.343,0.746
Tmin’		

Correction method= # Reported T Limits: Tmin=0.343 Tmax=0.746
AbsCorr = NONE

Data completeness= 0.995 Theta(max)= 28.283

R(reflections)= 0.0303(2067) wR2(reflections)= 0.0828(2281)

S = 1.048 Npar= 71

The following ALERTS were generated. Each ALERT has the format
test-name_ALERT_alert-type_alert-level.
Click on the hyperlinks for more details of the test.

Alert level C

```
PLAT042_ALERT_1_C Calc. and Reported MoietyFormula Strings  Differ     Please Check
              Calc: I4 Pb, 2(C4 H10 N)
              Rep.: I4 Pb, C8 H20 N2
PLAT053_ALERT_1_C Minimum Crystal Dimension Missing (or Error) ...     Please Check
PLAT054_ALERT_1_C Medium  Crystal Dimension Missing (or Error) ...     Please Check
PLAT055_ALERT_1_C Maximum Crystal Dimension Missing (or Error) ...     Please Check
PLAT342_ALERT_3_C Low Bond Precision on  C-C Bonds ..............     0.0105 Ang.
```

Alert level G

```
PLAT004_ALERT_5_G Polymeric Structure Found with Maximum Dimension          2 Info
PLAT005_ALERT_5_G No Embedded Refinement Details Found  in the CIF     Please Do !
PLAT007_ALERT_5_G Number of Unrefined Donor-H Atoms .............          3 Report
              H1A   H1B   H1C
PLAT199_ALERT_1_G Reported _cell_measurement_temperature ..... (K)        273 Check
PLAT200_ALERT_1_G Reported   _diffrn_ambient_temperature ..... (K)        273 Check
PLAT764_ALERT_4_G Overcomplete CIF Bond List Detected (Rep/Expd) .       1.18 Ratio
PLAT794_ALERT_5_G Tentative Bond Valency for Pb1       (II)      .       2.29 Info
```

0 **ALERT level A** = Most likely a serious problem - resolve or explain
0 **ALERT level B** = A potentially serious problem, consider carefully
5 **ALERT level C** = Check. Ensure it is not caused by an omission or oversight
7 **ALERT level G** = General information/check it is not something unexpected

6 ALERT type 1 CIF construction/syntax error, inconsistent or missing data
0 ALERT type 2 Indicator that the structure model may be wrong or deficient
1 ALERT type 3 Indicator that the structure quality may be low
1 ALERT type 4 Improvement, methodology, query or suggestion
4 ALERT type 5 Informative message, check

Datablock: WeichuanZhang_CDMA-N2

Bond precision:	C-C = 0.0500 A		Wavelength=0.71073
Cell:	a=8.8151(4)	b=8.7927(5)	c=36.583(2)
	alpha=90	beta=90	gamma=90
Temperature:	273 K		

	Calculated	Reported
Volume	2835.5(3)	2835.5(3)
Space group	P c a 21	P c a 21
Hall group	P 2c -2ac	P 2c -2ac
Moiety formula	I7 Pb2, C8 H20 N2, C H6 N	I7 Pb2, C8 H20 N2, C H6 N
Sum formula	C9 H26 I7 N3 Pb2	C9 H26 I7 N3 Pb2
Mr	1479.03	1479.01
Dx,g cm-3	3.465	3.465
Z	4	4
Mu (mm-1)	19.480	19.480
F000	2544.0	2544.0
F000'	2506.73	
h,k,lmax	10,10,43	9,10,43
Nref	5008[2550]	4760
Tmin,Tmax		0.303,0.746
Tmin'		

Correction method= # Reported T Limits: Tmin=0.303 Tmax=0.746
AbsCorr = MULTI-SCAN

Data completeness= 1.87/0.95 Theta(max)= 24.996

R(reflections)= 0.0596(4408) wR2(reflections)= 0.1766(4760)

S = 1.073 Npar= 194

The following ALERTS were generated. Each ALERT has the format
test-name_ALERT_alert-type_alert-level.
Click on the hyperlinks for more details of the test.

Alert level B

PLAT029_ALERT_3_B _diffrn_measured_fraction_theta_full value Low . 0.958 Why?

Author Response: The low completeness is due to the extremely large unit cell of perovskite structure,quality of the crystal, and geometrical constraints of the instrument, which leads to a data completeness lower than 100%.

PLAT213_ALERT_2_B Atom I2 has ADP max/min Ratio ..... 4.9 oblate

Author Response: It is caused by the slightly abnormal I2 and N3 thermal ellipsoid, which we have limited and refined, but still present.

```
PLAT213_ALERT_2_B Atom N3              has ADP max/min Ratio .....        5.0 oblate
```

Author Response: It is caused by the slightly abnormal I2 and N3 thermal ellipsoid, which we have limited and refined, but still present.

```
PLAT342_ALERT_3_B Low Bond Precision on  C-C Bonds ..............       0.05 Ang.
```

Author Response: The alarm are generated because the thermal ellipsoids of some carbon atoms are slightly larger and may have disordered structures, resulting in low bond precision on C-C bonds.

Alert level C

```
STRVA01_ALERT_4_C           Flack test results are ambiguous.
           From the CIF: _refine_ls_abs_structure_Flack    0.511
           From the CIF: _refine_ls_abs_structure_Flack_su    0.014
PLAT053_ALERT_1_C Minimum Crystal Dimension Missing (or Error) ...     Please Check
PLAT054_ALERT_1_C Medium  Crystal Dimension Missing (or Error) ...     Please Check
PLAT055_ALERT_1_C Maximum Crystal Dimension Missing (or Error) ...     Please Check
PLAT420_ALERT_2_C D-H Bond Without Acceptor  N        --Ha       .     Please Check
PLAT907_ALERT_2_C Flack x > 0.5, Structure Needs to be Inverted? .       0.51 Check
```

Alert level G

```
PLAT002_ALERT_2_G Number of Distance or Angle Restraints on AtSite         12 Note
PLAT003_ALERT_2_G Number of Uiso or Uij Restrained non-H Atoms ...         12 Report
PLAT004_ALERT_5_G Polymeric Structure Found with Maximum Dimension          2 Info
PLAT005_ALERT_5_G No Embedded Refinement Details Found  in the CIF     Please Do !
PLAT007_ALERT_5_G Number of Unrefined Donor-H Atoms .............          9 Report
              H2C    H2D    H2E    H3A    H3B    H3C    Ha     Hb     Hc
PLAT083_ALERT_2_G SHELXL Second Parameter in WGHT  Unusually Large     130.25 Why ?
PLAT111_ALERT_2_G ADDSYM Detects New (Pseudo) Centre of Symmetry .         95 %Fit
PLAT112_ALERT_2_G ADDSYM Detects New (Pseudo) Symm. Elem         n         95 %Fit
PLAT113_ALERT_2_G ADDSYM Suggests Possible Pseudo/New  Space Group       Pbcn Check
              Check Model Parameter Symmetry for Reflection Data Support
PLAT199_ALERT_1_G Reported _cell_measurement_temperature ..... (K)        273 Check
PLAT200_ALERT_1_G Reported   _diffrn_ambient_temperature ..... (K)        273 Check
PLAT232_ALERT_2_G Hirshfeld Test Diff (M-X)  Pb1      --I2_a     .        6.6 s.u.
PLAT720_ALERT_4_G Number of Unusual/Non-Standard Labels .........           3 Note
              Ha      Hb      Hc
PLAT764_ALERT_4_G Overcomplete CIF Bond List Detected (Rep/Expd) .       1.17 Ratio
PLAT794_ALERT_5_G Tentative Bond Valency for Pb1       (II)      .       2.30 Info
PLAT794_ALERT_5_G Tentative Bond Valency for Pb2       (II)      .       2.29 Info
PLAT860_ALERT_3_G Number of Least-Squares Restraints ............        204 Note
```

```
   0 ALERT level A = Most likely a serious problem - resolve or explain
   4 ALERT level B = A potentially serious problem, consider carefully
   6 ALERT level C = Check. Ensure it is not caused by an omission or oversight
  17 ALERT level G = General information/check it is not something unexpected

   5 ALERT type 1 CIF construction/syntax error, inconsistent or missing data
```

11 ALERT type 2 Indicator that the structure model may be wrong or deficient
3 ALERT type 3 Indicator that the structure quality may be low
3 ALERT type 4 Improvement, methodology, query or suggestion
5 ALERT type 5 Informative message, check

Datablock: WeichuanZhang_CDMA-N3

Bond precision: C-C = 0.0625 A Wavelength=0.71073

Cell:	a=8.8042(15)	b=8.8303(16)	c=48.841(8)
	alpha=90	beta=90	gamma=90
Temperature:	273 K		

	Calculated	Reported
Volume	3797.1(11)	3797.1(11)
Space group	P n a 21	P n a 21
Hall group	P 2c -2n	P 2c -2n
Moiety formula	I10 Pb3, C8 H20 N2, 2(C H6 N)	I10 Pb3, 2(C H6 N), C8 H20 N2
Sum formula	C10 H32 I10 N4 Pb3	C10 H32 I10 N4 Pb3
Mr	2099.00	2098.96
Dx,g cm-3	3.672	3.672
Z	4	4
Mu (mm-1)	21.407	21.407
F000	3584.0	3584.0
F000’	3529.15	
h,k,lmax	10,10,58	10,10,58
Nref	6711[3399]	6615
Tmin,Tmax		0.095,0.262
Tmin’		

Correction method= # Reported T Limits: Tmin=0.095 Tmax=0.262
AbsCorr = NONE

Data completeness= 1.95/0.99 Theta(max)= 24.998

R(reflections)= 0.0835(4757) wR2(reflections)= 0.2491(6615)

S = 1.031 Npar= 250

```
The following ALERTS were generated. Each ALERT has the format
       test-name_ALERT_alert-type_alert-level.
Click on the hyperlinks for more details of the test.
```

Alert level B

```
PLAT250_ALERT_2_B Large U3/U1 Ratio for <U(i,j)> Tensor(Resd    2)        5.8 Note
```

Author Response: Because the thermal ellipsoids of some atomic temperature factors are not close to spherical, the difference between the directions of U3 and U1 is large.

```
PLAT342_ALERT_3_B Low Bond Precision on  C-C Bonds ...............     0.0625 Ang.
```

Author Response: Because the thermal ellipsoids of some carbon atoms are slightly larger and may have disordered structures, resulting in low bond precision on C-C bonds.

Alert level C

```
STRVA01_ALERT_4_C           Flack test results are ambiguous.
           From the CIF: _refine_ls_abs_structure_Flack    0.420
           From the CIF: _refine_ls_abs_structure_Flack_su    0.040
PLAT042_ALERT_1_C Calc. and Reported MoietyFormula Strings  Differ     Please Check
              Calc: I10 Pb3, C8 H20 N2, 2(C H6 N)
              Rep.: I10 Pb3, 2(C H6 N), C8 H20 N2
PLAT053_ALERT_1_C Minimum Crystal Dimension Missing (or Error) ...     Please Check
PLAT054_ALERT_1_C Medium  Crystal Dimension Missing (or Error) ...     Please Check
PLAT055_ALERT_1_C Maximum Crystal Dimension Missing (or Error) ...     Please Check
PLAT214_ALERT_2_C Atom N2        (Anion/Solvent) ADP max/min Ratio        4.2 oblate
PLAT242_ALERT_2_C Low    'MainMol' Ueq as Compared to Neighbors of        Pb1 Check
PLAT414_ALERT_2_C Short Intra D-H..H-X       H4C      ..H6B      .       1.93 Ang.
                                                  x,y,z  =      1_555 Check
PLAT420_ALERT_2_C D-H Bond Without Acceptor  N1       --H1D      .     Please Check
PLAT420_ALERT_2_C D-H Bond Without Acceptor  N1       --H1E      .     Please Check
PLAT420_ALERT_2_C D-H Bond Without Acceptor  N1       --H1F      .     Please Check
PLAT420_ALERT_2_C D-H Bond Without Acceptor  N2       --H2D      .     Please Check
PLAT420_ALERT_2_C D-H Bond Without Acceptor  N2       --H2E      .     Please Check
PLAT420_ALERT_2_C D-H Bond Without Acceptor  N2       --H2F      .     Please Check
PLAT420_ALERT_2_C D-H Bond Without Acceptor  N3       --H3D      .     Please Check
PLAT420_ALERT_2_C D-H Bond Without Acceptor  N4       --H4C      .     Please Check
```

Alert level G

```
PLAT002_ALERT_2_G Number of Distance or Angle Restraints on AtSite         12 Note
PLAT003_ALERT_2_G Number of Uiso or Uij Restrained non-H Atoms ...         27 Report
PLAT004_ALERT_5_G Polymeric Structure Found with Maximum Dimension          2 Info
PLAT005_ALERT_5_G No Embedded Refinement Details Found  in the CIF     Please Do !
PLAT007_ALERT_5_G Number of Unrefined Donor-H Atoms ..............         12 Report
              H3C   H3D   H3E   H4A   H4B   H4C   H1D   H1E   H1F   H2D   H2E
              H2F
PLAT072_ALERT_2_G SHELXL First  Parameter in WGHT  Unusually Large       0.12 Report
```

```
PLAT083_ALERT_2_G SHELXL Second Parameter in WGHT  Unusually Large     103.95 Why ?
PLAT111_ALERT_2_G ADDSYM Detects New (Pseudo) Centre of Symmetry .        100 %Fit
PLAT112_ALERT_2_G ADDSYM Detects New (Pseudo) Symm. Elem        m          92 %Fit
PLAT113_ALERT_2_G ADDSYM Suggests Possible Pseudo/New  Space Group       Pnma Check
              Check Model Parameter Symmetry for Reflection Data Support
PLAT199_ALERT_1_G Reported _cell_measurement_temperature ..... (K)        273 Check
PLAT200_ALERT_1_G Reported   _diffrn_ambient_temperature ..... (K)        273 Check
PLAT764_ALERT_4_G Overcomplete CIF Bond List Detected (Rep/Expd) .       1.20 Ratio
PLAT794_ALERT_5_G Tentative Bond Valency for Pb1       (II)      .       2.39 Info
PLAT794_ALERT_5_G Tentative Bond Valency for Pb2       (II)      .       2.26 Info
PLAT794_ALERT_5_G Tentative Bond Valency for Pb3       (II)      .       2.43 Info
PLAT860_ALERT_3_G Number of Least-Squares Restraints ............         157 Note
```

```
  0 ALERT level A = Most likely a serious problem - resolve or explain
  2 ALERT level B = A potentially serious problem, consider carefully
 16 ALERT level C = Check. Ensure it is not caused by an omission or oversight
 17 ALERT level G = General information/check it is not something unexpected

  6 ALERT type 1 CIF construction/syntax error, inconsistent or missing data
 19 ALERT type 2 Indicator that the structure model may be wrong or deficient
  2 ALERT type 3 Indicator that the structure quality may be low
  2 ALERT type 4 Improvement, methodology, query or suggestion
  6 ALERT type 5 Informative message, check
```

It is advisable to attempt to resolve as many as possible of the alerts in all categories. Often the minor alerts point to easily fixed oversights, errors and omissions in your CIF or refinement strategy, so attention to these fine details can be worthwhile. In order to resolve some of the more serious problems it may be necessary to carry out additional measurements or structure refinements. However, the purpose of your study may justify the reported deviations and the more serious of these should normally be commented upon in the discussion or experimental section of a paper or in the "special_details" fields of the CIF. checkCIF was carefully designed to identify outliers and unusual parameters, but every test has its limitations and alerts that are not important in a particular case may appear. Conversely, the absence of alerts does not guarantee there are no aspects of the results needing attention. It is up to the individual to critically assess their own results and, if necessary, seek expert advice.

Publication of your CIF in IUCr journals

A basic structural check has been run on your CIF. These basic checks will be run on all CIFs submitted for publication in IUCr journals (*Acta Crystallographica*, *Journal of Applied Crystallography*, *Journal of Synchrotron Radiation*); however, if you intend to submit to *Acta Crystallographica Section C* or *E* or *IUCrData*, you should make sure that full publication checks are run on the final version of your CIF prior to submission.

Publication of your CIF in other journals

Please refer to the *Notes for Authors* of the relevant journal for any special instructions relating to CIF submission.

PLATON version of 06/01/2024; check.def file version of 05/01/2024

Datablock WeichuanZhang_CDMA-N1 - ellipsoid plot

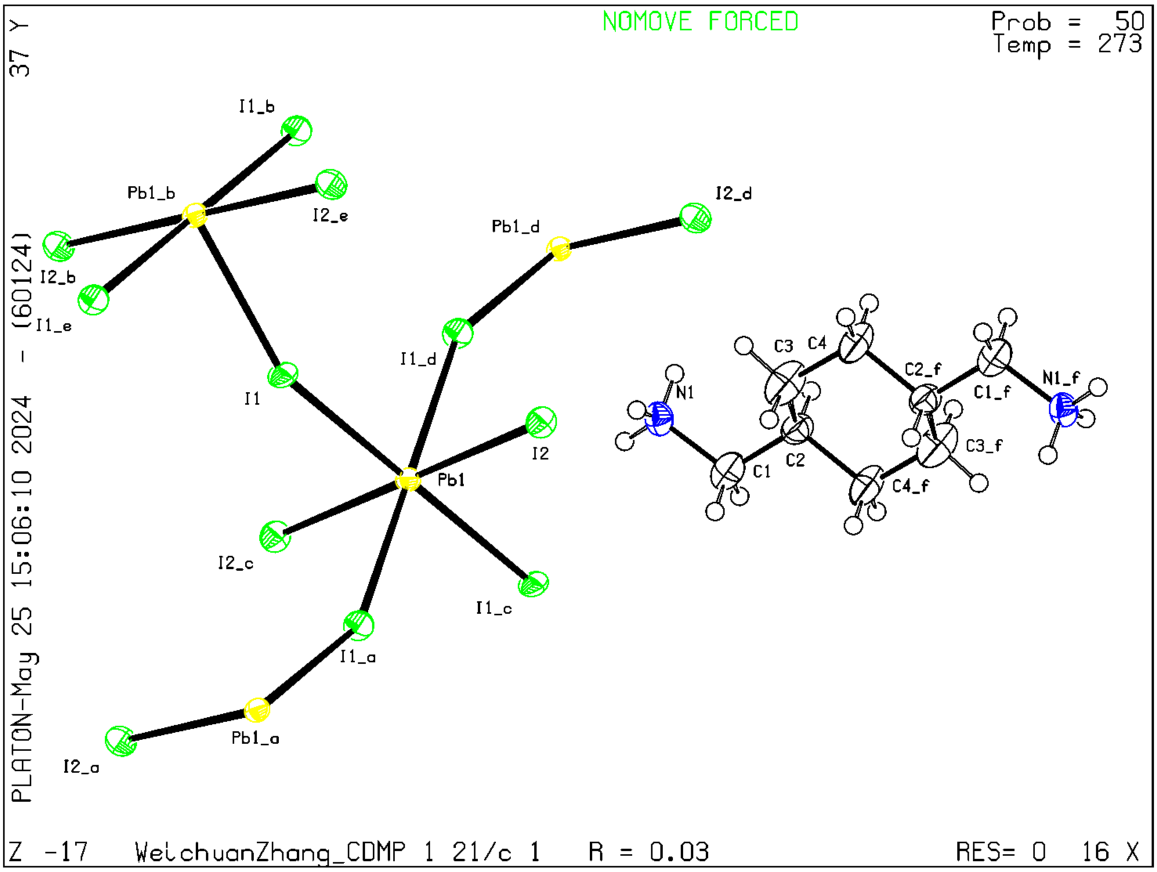

Datablock **WeichuanZhang_CDMA-N2** - ellipsoid plot

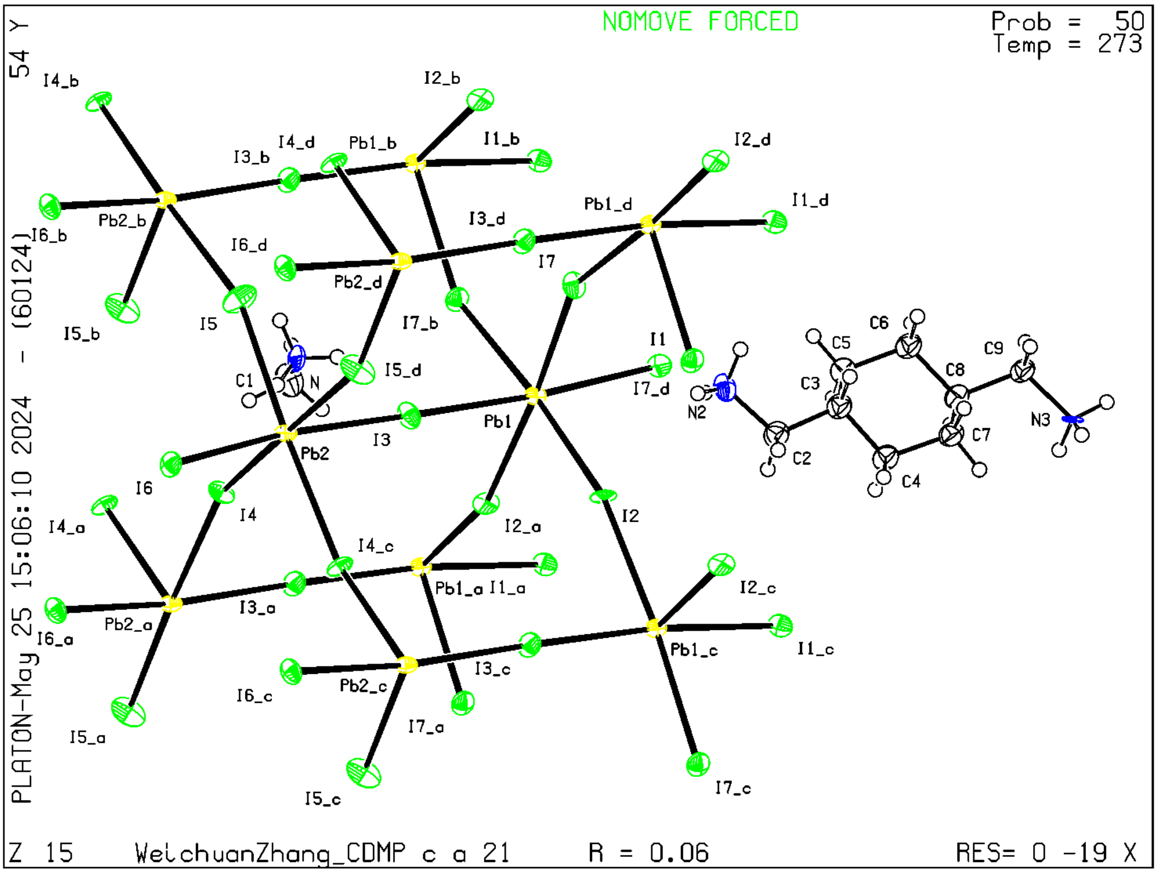

Datablock WeichuanZhang_CDMA-N3 - ellipsoid plot

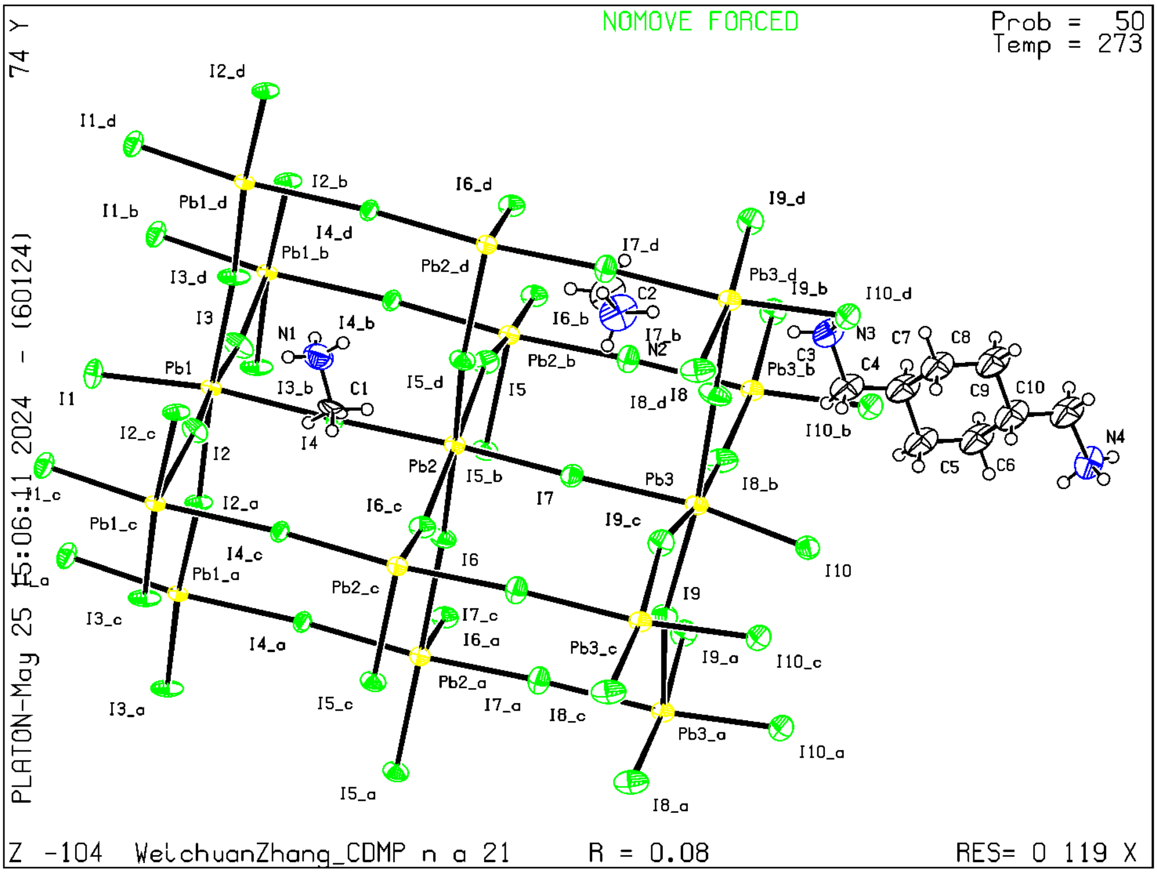

Supplement: Supplementary file 5 — Supplementary Data 2 [file 41467_2024_50018_MOESM5_ESM.pdf]
