# Supplementary material for: Ultrastable and efficient slight-interlayer-displacement 2D Dion-Jacobson perovskite solar cells
Source: Nat Commun. 2024 Jul 8;15:5709. doi: 10.1038/s41467-024-50018-4 (PMC11231157; doi:10.1038/s41467-024-50018-4)
Supplement: Supplementary file 9 — Reporting Summary [file 41467_2024_50018_MOESM9_ESM.pdf]

nature portfolio

Corresponding author(s): Huiqiong Zhou

Solar Cells Reporting Summary

Nature Portfolio wishes to improve the reproducibility of the work that we publish. This form is intended for publication with all accepted papers reporting the characterization of photovoltaic devices and provides structure for consistency and transparency in reporting. Some list items might not apply to an individual manuscript, but all fields must be completed for clarity.

For further information on Nature Research policies, including our data availability policy, see Authors & Referees.

Experimental design

Please check the following details are reported in the manuscript, and provide a brief description or explanation where applicable.

1. Dimensions

Item	Reported	Description / Explanation
Area of the tested solar cells	☒ Yes ☐ No	0.04 cm2 / *Explain why this information is not reported/not relevant.*
Method used to determine the device area	☒ Yes ☐ No	Defined by the shadow masks / *Explain why this information is not reported/not relevant.*

2. Current-voltage characterization

Item	Reported	Description / Explanation
Current density-voltage (J-V) plots in both forward and backward direction	☐ Yes ☒ No	Hysteresis statistics for data processing through forward and backward scanning are shown in Fig. 2c.
Voltage scan conditions	☒ Yes ☐ No	Please see Fig 1b, 2b, Supplementary Fig. 3 and the chapter of Methods. Devices were measured in a reverse scan (1.30 V to -0.1 V with steps of 0.02 V) / *Explain why this information is not reported/not relevant.*
Test environment	☒ Yes ☐ No	Devices were measured at room temperature (about 298.15K) in N2-filled glove box, described in the section of "Methods" in the Manuscript. / *Explain why this information is not reported/not relevant.*
Protocol for preconditioning of the device before its characterization	☐ Yes ☒ No	*Provide a description of the protocol.* / No preconditioning protocol.
Stability of the J-V characteristic	☒ Yes ☐ No	Stability of the J-V characteristic were obtained by tracking the maximum power point. Please see Fig 1e. / *Explain why this information is not reported/not relevant.*

3. Hysteresis or any other unusual behaviour

Item	Reported	Description / Explanation
Description of the unusual behaviour observed during the characterization	☒ Yes ☐ No	No unusual behavior was found. The target perovskite solar cells have a relatively lower and stable-distributed hysteresis. / *Explain why this information is not reported/not relevant.*
Related experimental data	☒ Yes ☐ No	Please see Fig 2c. / *Explain why this information is not reported/not relevant.*

4. Efficiency

Item	Reported	Description / Explanation
External quantum efficiency (EQE) or incident photons to current efficiency (IPCE)	☒ Yes ☐ No	Please see Supplementary Fig. 4, Supplementary Fig. 5, and experimental methods for details. / *Explain why this information is not reported/not relevant.*
A comparison between the integrated response under the standard reference spectrum and the response measure under the simulator	☒ Yes ☐ No	Please see main text. The integrated JSC values is matched well with those measured J-V curves (Less than 5%). / *Explain why this information is not reported/not relevant.*

For tandem solar cells, the bias illumination and bias voltage used for each subcell

☐ Yes — *Provide a description of the measurement conditions.*
☒ No — Not relevant.

5. Calibration

Light source and reference cell or sensor used for the characterization

☒ Yes — Please see the chapter of Methods.
☐ No — *Explain why this information is not reported/not relevant.*

Confirmation that the reference cell was calibrated and certified

☒ Yes — Please see the chapter of Methods.
☐ No — *Explain why this information is not reported/not relevant.*

Calculation of spectral mismatch between the reference cell and the devices under test

☒ Yes — The light spectrum used for measurements matches well with the reference silicon cell. And no spectral mismatch calculation was performed in our lab.
☐ No — *Explain why this information is not reported/not relevant.*

6. Mask/aperture

Size of the mask/aperture used during testing

☒ Yes — 0.04 cm2
☐ No — *Explain why this information is not reported/not relevant.*

Variation of the measured short-circuit current density with the mask/aperture area

☐ Yes — *Report the difference in the short-circuit current density values measured with the mask and aperture area.*
☒ No — The mask/aperture area is fixed and cannot change.

7. Performance certification

Identity of the independent certification laboratory that confirmed the photovoltaic performance

☐ Yes — *Identify the independent certification laboratory.*
☒ No — Not relevant to this work. The work focused on stability and did not break efficiency records enough to require certification.

A copy of any certificate(s)

☐ Yes — *Certificate copies should be provided in the Supplementary information. Please state the supplementary item number.*
☒ No — The claim of efficiency certification is beyond the scope of this work.

8. Statistics

Number of solar cells tested

☒ Yes — 20 devices
☐ No — *Explain why this information is not reported/not relevant.*

Statistical analysis of the device performance

☒ Yes — Please see Fig 2a (inset) and Fig 2c .
☐ No — *Explain why this information is not reported/not relevant.*

9. Long-term stability analysis

Type of analysis, bias conditions and environmental conditions

☒ Yes — The thermal stability of the non-encapsulated solar cell was tested in a nitrogen atmosphere by tracking the device performance at about 85 °C. Fractional devices were aged under open-circuit conditions, and were taken out from the chamber and tested at different time intervals. Perovskite solar cells were measured under illumination by an Air Mass 1.5 Global (AM 1.5) solar simulator (Enli Technology Co., Ltd.) with an irradiation intensity of 100 mW cm−2. When measuring the cells during ageing, we removed them from the ageing chamber and allowed them to cool to room temperature, which typically took a few minutes. For the humidity stability, the non-encapsulated perovskite films was store in a constant temperature humidity chamber and were taken out from the chamber and tested at different time intervals. The relative humidity in the chamber was within 90 ± 3% during the entire ageing test. Operational stability of unencapsulated devices under a white light-emitting diode lamp at maximum power point in a nitrogen-filled glove box. The measured time interval was two hours. Please see Fig 1.
☐ No — *Explain why this information is not reported/not relevant.*
